# Ligand-Assisted Colloidal Synthesis of Alkali Metal-Based Ternary Chalcogenide: Nanostructuring and Phase Control in Na–Cu–S System

**DOI:** 10.1021/acs.nanolett.4c04257

**Published:** 2025-03-12

**Authors:** Hannah McKeever, Nilotpal Kapuria, Adair Nicolson, Suvodeep Sen, David Scanlon, Kevin M. Ryan, Shalini Singh

**Affiliations:** † Department of Chemical Sciences and Bernal Institute, University of Limerick, V94 T9PX Limerick, Ireland; ‡ Department of Chemistry, Indiana University − Bloomington, 800 East Kirkwood Avenue, Bloomington, Indiana 47405, United States; § School of Chemistry, University of Birmingham, Birmingham B15 2TT, United Kingdom; ∥ Department of Chemistry, University College London, London WC1H 0AJ, United Kingdom

**Keywords:** ternary chalcogenides, colloidal nanocrystals, energy materials, alkali metal chalcogenide, phase control

## Abstract

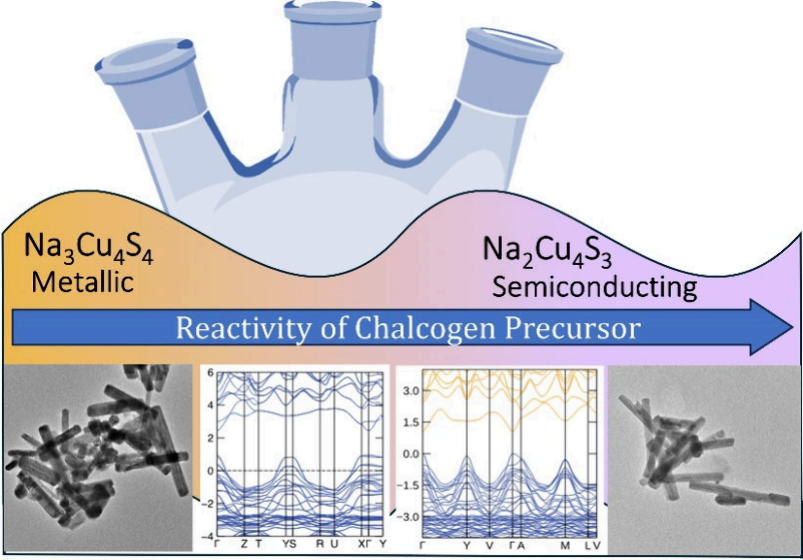

The development of sustainable and tunable materials is crucial for advancing modern technologies. We present a controlled synthesis of colloidal Na–Cu–S nanostructures. To overcome the reactivity difference between Na and Cu precursors toward chalcogens in a colloidal synthesis and to achieve metastable phase formation, we designed a single-source precursor for Cu and S. The decomposition of this precursor creates a Cu–S template into which Na ions diffuse to form metastable Na–Cu–S. By leveraging the reactivity of sulfur precursors, we synthesized Na_3_Cu_4_S_4_ (orthorhombic) and Na_2_Cu_4_S_3_ (monoclinic) nanocrystals with distinct properties. A mechanistic investigation reveals a predictive pathway previously unobserved in alkali-metal-based ternary chalcogenide systems. Further, computational DFT calculations demonstrate that Na_3_Cu_4_S_4_ exhibits metallic characteristics while Na_2_Cu_4_S_3_ is semiconducting, with an optimal band gap for photovoltaic applications. This research advances our understanding of ternary chalcogenide systems and establishes a framework for the rational design of complex nanomaterials.

A wide array of current technologies is underpinned by critical and nonsustainable material compositions. This has motivated the research and development of analogous materials with sustainable elemental compositions and comparable or enhanced properties. In this context, a group of alkali metal-based ternary chalcogenides (ABZ compounds where A = alkali metal, B = transition metals/pnictogens, Z = chalcogen) has been highlighted by computational and experimental studies as suitable sustainable compounds with multifunctional applications across optoelectronics, thermoelectrics, and energy storage.^1−4^ Experimentally synthesized ABZ materials which have shown technological potential include NaSbZ_2_, LiSbS_2_, KCuZ, NaBiZ_2_, and CsCu_5_Z_3_ (Z = S, Se).^5−11^ They have a wide range of charge states, giving each ternary system a complex phase diagram with multiple stoichiometries and varied properties to discover. Sodium copper sulfide is one such example from this class of materials with a sustainable and benign composition. Computational simulations have predicted the existence of various stoichiometries existing in a range of crystalline phases. For instance, NaCu_4_S_4_ (trigonal), Na_3_Cu_4_S_4_ (orthorhombic),^12,13^ and Na_4_Cu_2_S_3_ (tetragonal) are predicted to be stable, while NaCu_4_S_3_ (trigonal), Na_2_Cu_4_S_3_ (monoclinic), Na_7_Cu_12_S_10_ (monoclinic),^14^ and NaCu_5_S_3_ (hexagonal)^15^ are considered unstable or metastable.^16^ From a synthesis standpoint, the closest materialization of the Na–Cu–S system has been the recent reports on producing mixed valent NaCu_4_S_4_ and NaCu_4_S_3_ as two-dimensional metallic materials.^17,18^ The solid-state synthesis was employed at temperatures ranging from 600–800 °C for >12 h. These Na-deficient crystals exhibited low control over phase purity.^18^ A significant bottleneck exists in achieving phase purity or stabilization of the metastable phases for Na–Cu–S through conventional solid-state reactions. The precise compositional tunability in the crystal forms can be restricted by the high reactivity of reduced forms of Na metal during higher temperature syntheses or the high mobility of Na cations in the final ternary compounds.

In such a scenario, colloidal chemistry-based approaches can become a facile and adaptable synthetic platform to explore the vast compositional landscape of Na–Cu–S. Colloidal chemistry can offer exquisite control over dimension, form, and composition through the interplay of precursor–ligand–temperature. This enables access to metastable phases and facilitates precise control over the nature and extent of interfaces between distinct crystal domains, which extends the range of unique properties that are relevant from a technological viewpoint.

Driven by the synthetic limitations that restrict the ability to explore the complex compositional landscape of the Na–Cu–S system, we present a colloidal chemistry-based synthesis of Na–Cu–S nanocrystals (NCs) with controlled stoichiometries, crystalline phases, and varied properties. Due to the disparity in reactivity between Na and Cu precursors toward chalcogens, the synthesis was initiated by designing carbamate-based single-source precursors (SSP) for Cu and S. The in situ decomposition of SSP in the reaction flask produced a Cu–S-based template of a layered structure that facilitated the accommodation of Na ions in the lattice to generate the metastable Na–Cu–S NCs. Furthermore, control over the phase and stoichiometries of Na–Cu–S was achieved by utilizing the reactivity of sulfur precursors based on their bond dissociation energies (BDE). Through detailed structural analysis, we show the formation of phase pure Na_3_Cu_4_S_4_ (orthorhombic) and Na_2_Cu_4_S_3_ (monoclinic) NCs. The DFT electronic structure calculations demonstrated that Na_3_Cu_4_S_4_ is metallic while Na_2_Cu_4_S_3_ is semiconducting in nature with an optimal direct band gap for photovoltaic applications. The precisely controlled synthesis, combined with an in-depth mechanistic investigation, reveals a predictive pathway for NC formation, previously not observed in ABZ systems.

Na_3_Cu_4_S_4_ is hereafter referred to as NaCuS-(O), and Na_2_Cu_4_S_3_ is hereafter referred to as NaCuS-(M) (Table S1 in Supporting Information (SI)). NCs were synthesized via a colloidal hot injection method (see SI, sections 2–8 for detailed synthesis and characterization procedures). For the synthesis, sodium oleate and copper diethyldithiocarbamate (Cu–DDTC) were used as cationic precursors, with oleylamine (OLA) as both a solvent and reducing agent. Throughout the reaction process, all parameters were kept constant, with the only variation being the type of sulfur source injected. The reactivity of the sulfur sources, 1-dodecanethiol (DDT) and *tert*-butyl disulfide (TBDS), determines the formation of two distinct compositional NCs. Specifically, a TBDS injection produces NaCuS-(O) NCs, and a DDT injection produces NaCuS-(M) NCs.

Powder X-ray diffraction (XRD) analysis was used to determine the crystal structure of the as-synthesized NCs. From the XRD in Figure 1a and d, it was deduced that NaCuS-(O) crystallizes into the orthorhombic *Pbam* (55) space group, while NaCuS-(M) was found to crystallize in a monoclinic system, the *C*2/*m* (12) space group. Rietveld refinement of the XRD patterns in Figures S1 and S2 proves that the lattice parameters of both NaCuS-(O) and NaCuS-(M) are in good agreement with the reported values. Low magnification transmission electron microscopy (TEM) images in Figure 1b and e show both NC compositions as cuboid in shape. Both NC systems possess lengths in the microsize range with wide size distribution, with the widths having an average size of ∼59.7 and ∼52.5 nm for NaCuS-(O) (Figure S3) and NaCuS-(M) (Figure S4), respectively. High-resolution TEM (HRTEM) analysis provided additional confirmation of the successful formation of NaCuS-(O) and NaCuS-(M). From the HRTEM analysis of NaCuS-(O) in Figure 1c, the fast Fourier transform (FFT) shows *d*-spacing of ∼3.1 and ∼2.9Å for the (111) and (020) planes, respectively, with an angle of ∼61.6° between the planes. In Figure 1f, the *d*-spacing values calculated for (601) and (021)planes of NaCuS-(M) are ∼3.9 and ∼5.1Å, respectively, with the measured angle of ∼90.6° between them. The *d*-spacing values and angles measured here closely match the reference values reported. Scanning TEM-energy dispersive spectroscopy (STEM-EDS) elemental mapping (Figures S5 and S6) further confirms the presence and homogeneous distribution of Na, Cu, and S in both NC systems.

**Figure 1:**
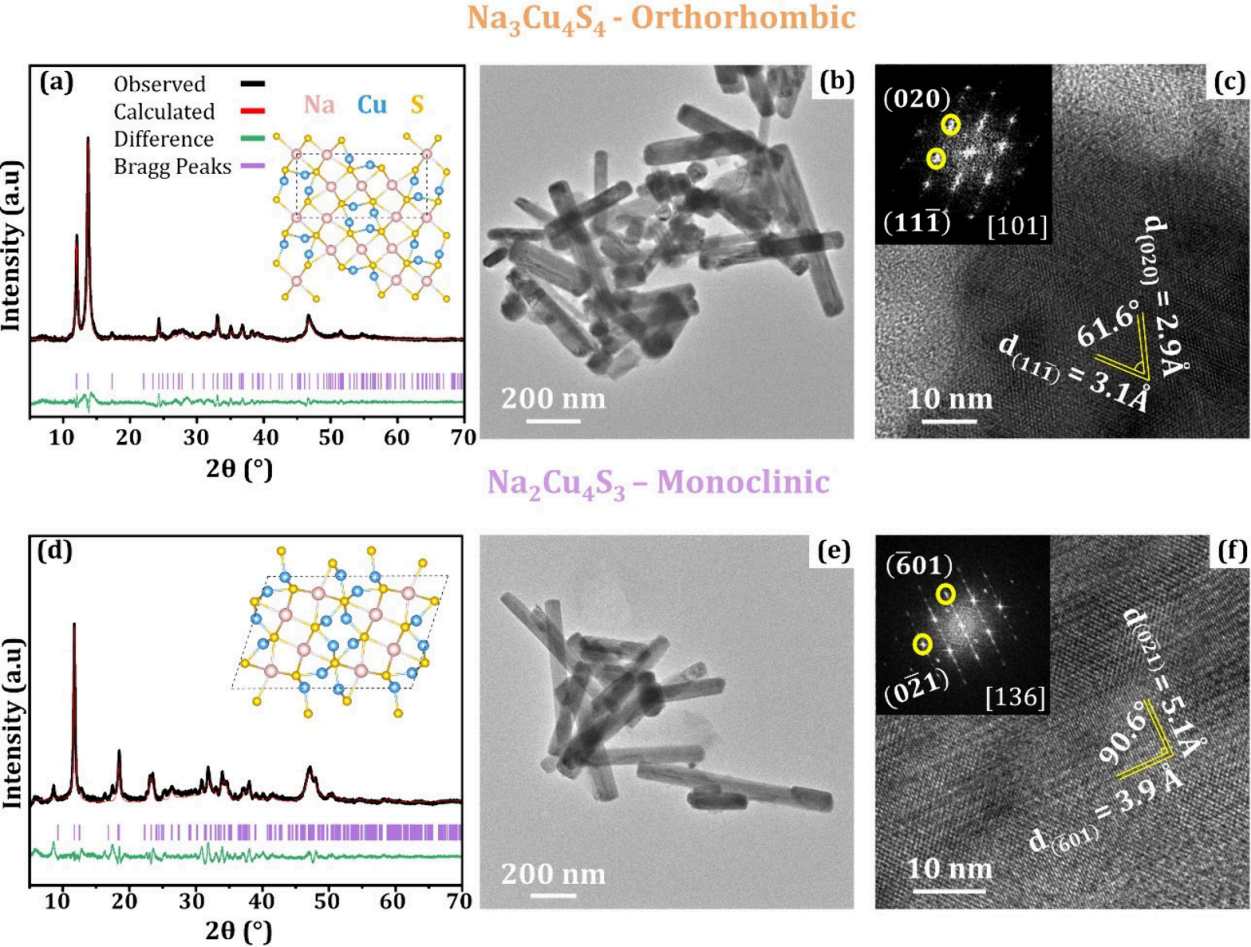
Characterization of Na_3_Cu_4_S_4_ (a–c) and Na_2_Cu_4_S_3_ (d–f). (a, d) X-ray diffraction patterns with reference patterns and crystal structures in the inset. (b, e) TEM and (c, f) HRTEM with selected area FFT patterns in the insets.

A detailed picture of the elemental composition, chemical, and electronic state of the elements in NaCuS-(O) and NaCuS-(M) was obtained by X-ray photoelectron spectroscopy (XPS) analysis (Figure 2a–c and Figure S7). For both Na–Cu–S compositions, XPS analysis shows a peak at ∼1071.5 eV corresponding to the presence of Na^+^ (Figure 2a). The Cu 2p spectra in Figure 2b exhibit a single doublet peak with 2p_3/2_ at ∼932 eV which corresponds to the Cu^+^ species, indicating that copper has an oxidation state of +1. In NaCuS-(M), there is no evidence of Cu^2+^ due to the lack of satellite peaks in the high-resolution Cu spectra; this can be determined by the fact that Cu^2+^ compounds show satellite peaks at ∼945 eV while Cu^+^ compounds do not.^19^ In NaCuS-(O), there is a slight hump at ∼945 eV which suggests the possibility of a very small amount of Cu^2+^. The mixed valence of Na_3_Cu_4_S_4_ has been discussed, but our results show that based on peak ratios and fitting the system is mainly composed of Cu^+^.^12^ The sulfur spectra in Figure 2c showed 4 doublets during peak fitting. The first, at binding energy 160.5 eV, is characteristic of the sulfide bond (S^2–^), and the second, which is more intense, at 161.5 eV is also characteristic of a sulfide. The minor doublet peak at 160.8 eV corresponds to residue thiols, and the final higher energy doublet is characteristic of a sulfate (SO_4_^2–^), which shows that a certain degree of oxidation has occurred to a trace amount of sulfur. The ratio of peak areas shows that the concentrations of thiol and sulfate species are low, suggesting they are a minority in the sample compared with the sulfide species, which dominates both samples. The surface functionalities of the synthesized NCs were analyzed by using Fourier transform infrared spectroscopy (FTIR), as illustrated in Figure 2d. The FTIR shows that both OLA and dithiocarbamate (DTC) are bound to NaCuS-(O) and NaCuS-(M) NCs. The OLA exhibits its characteristic peaks at ∼1130 cm^–1^ (C–N stretching), 1600 cm^–1^ (N–H bending), and a broad stretching band around ∼3350 cm^–1^ arising due to a NH_2_ stretching vibration.^20,21^ In the case of the DTC ligands, there are three characteristic stretching vibrations, all of which are evident in the spectra. Strong (C–S) bands are present between 800 and 960 cm^–1^; the two bands observed in this region can be attributed to both asymmetric and symmetric vibrations that arise from the bidentate nature of the DTC ligand.^22^ The (C–N) stretching for DTC can be observed at 1498 cm^–1^, and the characteristic (C=S) stretching can be observed by the bands at 1380, 1464, and 1494 cm^–1^.^23^

**Figure 2:**
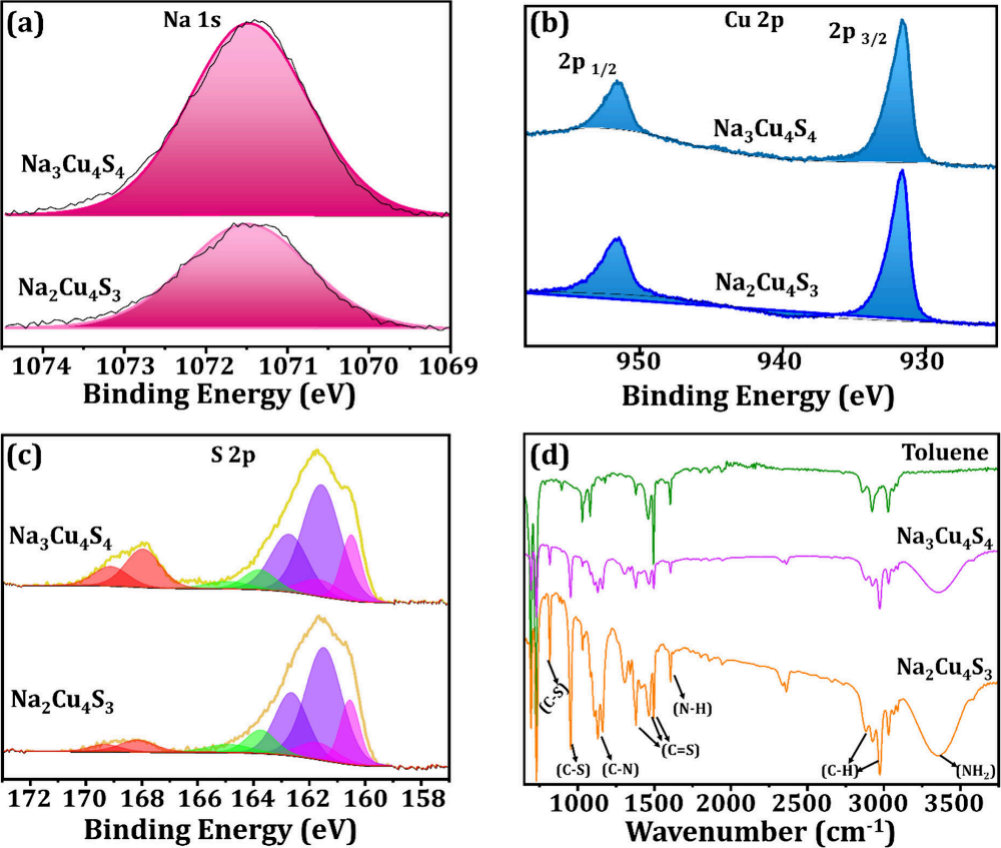
XPS and FTIR spectra for NaCuS-(O) and NaCuS-(M). (a) High-resolution XPS spectra of Na 1S. (b) High resolution XPS spectra of Cu 2p. (c) High resolution XPS spectra of S 2p. (d) FTIR spectra with toluene for reference.

To gain insight into the electronic and optical properties, hybrid density functional theory (DFT) calculations were performed on both NaCuS-(O) and NaCuS-(M) (see Supporting Information for extended methods).^24−32^ In agreement with previous reports, we found NaCuS-(O) to be metallic in nature (Figure S8).^12,13^ NaCuS-(M) is found to be a semiconducting material with a calculated optical band gap of 1.79 eV (Figure S9). Using the HSE06 functional, NaCuS-(M) was calculated to have a direct band gap of 1.07 eV at the G point (Figure 3a, b), and the orbital decomposed density of states show that the valence band is dominated by S p and Cu d states, while the conduction band is made up of Cu s and S p states. To be a viable thin film photovoltaic absorber, strong optical absorption is a requirement, with the absorption coefficient ideally reaching a value greater than 10^4^ cm^–1^.^33^ Due to the orbital makeup of the band edges, NaCuS-(M) has a delayed absorption onset due to the angular momentum selection rule (*Δl* = ± 1). Through orbital mixing at the band edges, the transition at the fundamental gap is only weakly forbidden, which, when combined with the low joint density of states due to the dispersive band edges, results in the absorption coefficient remaining low until above 1.6 eV and reaching 10^5^ cm^–1^ above 2.6 eV. This results in a maximum efficiency calculated using the spectroscopic limited maximum efficiency (SLME) metric of 24% for a film of 500 nm (common photovoltaic thickness), dropping to 6% for a film with a thickness of 30 nm (ultrathin solar cells, e.g., AgBiS_3_).^34^ This highlights NaCuS-(M)’s potential as a nontoxic/earth-abundant photovoltaic absorber, although it is unsuitable for ultrathin devices. These findings provide insight into the absorbance of NaCuS-(O) and NaCuS-(M), but further experimental research is needed to explore their optical behaviors in detail.

**Figure 3:**
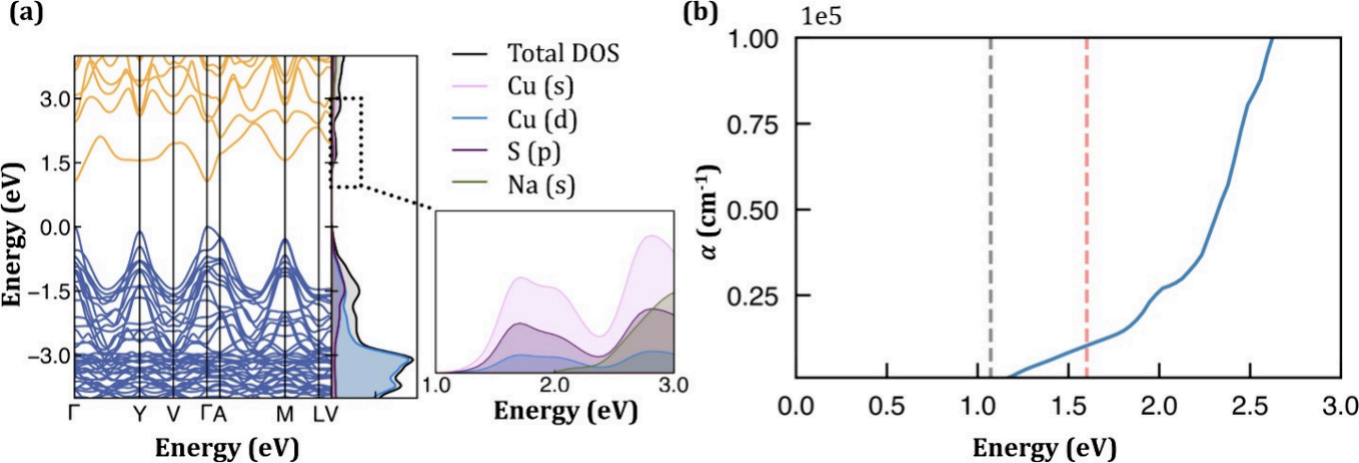
(a) Electronic band structure alongside the total and orbital-decomposed density of states calculated using HSE06 (Eg = 1.07 eV) for NaCuS-(M); valence band in blue, conduction band in orange, and valence band maximum (VBM) set to 0 eV for NaCuS-(M). The total density of states is not shown in the inset to aid in distinguishing between the orbital contributions. (b) Calculated band–band optical absorption with the fundamental bandgap marked by the grey dashed line. The red dashed line marks the point where the absorption coefficient is greater than 10^4^ cm^–1^.

The highly dispersive band edges lead to low effective masses for both electrons and holes, which are shown in Table 1, indicating the possibility for highly mobile carriers. The valence band shows significantly more anisotropy with a large effective mass of 1.34 *m*_*e*_ between Γ → *A* and a lower effective mass of 0.16 from Γ → *V*/*A*. This anisotropy, combined with the near band degeneracy at *A*, *Y*, and *V*, can lead to a high Seebeck coefficient through increasing the density of states effective mass, without significantly reducing the conductivity, making NaCuS-(M) a potential thermoelectric material.^35−37^

**Table 1:** Calculated Carrier Effective Masses for NaCuS-(M) Using HSE06 Hybrid DFT^a^

Valence Band (*m*_*e*_)			Conduction Band (*m*_*e*_)		
(Γ → *Y*)	(Γ → *V*)	(Γ → *A*)	(Γ → *Y*)	(Γ → *V*)	(Γ → *A*)
0.16	0.17	1.34	0.27	0.27	0.33

a The k-path is given in parentheses.

To gain mechanistic insights into the control over phase and stoichiometry in the colloidal Na–Cu–S system, aliquots of the reaction solution were analyzed via XRD, TEM, and STEM-EDS techniques (Figure 4a–h and Figures S10–S12). Initially, primary nucleation occurs to form hexagonal CuS through the thermal decomposition of the Cu-DDTC precursor in the OLA (Figure S11). This SSP acts as both the copper and sulfur source.^38^ The thermolysis of the disulfide bond in CuS at 280 °C in the presence of the reducing agent OLA directs the thermodynamically driven phase transition from hexagonal CuS to trigonal Cu_9_S_5_.^39^ This thermodynamically controlled change in crystallography from hexagonal to trigonal structure aligns well with previous reports on CuS phase transitions.^40−43^ The reaction is continued to be subjected to a heating phase and heating from 280 to 300 °C facilitates the formation of orthorhombic NaCuS-(O) nanoparticles alongside Cu_9_S_5_. The XRD in Figure 4a further confirms the presence of both Cu_9_S_5_ and NaCuS-(O). As the temperature increases toward 320 °C, the Cu_9_S_5_ phase transforms into NaCuS-(M), through their similar crystallographic structures of a hexagonal close-packed (hcp) sulfur sublattice. As seen in Figure 4a, NaCuS-(M) appears alongside NaCuS-(O) and Cu_9_S_5_ in the XRD spectra. Copper sulfide has been widely reported as a template for ternary compositions through cation exchange and other modification processes such as diffusion and migration, and we propose Cu_9_S_5_ to be the template for the formation of the NaCuS-(M) material.^40,44,45^ We hypothesize that SSP, Cu-DDTC initially provides a limiting amount of sulfur in the system. The high mobility of Cu ions in the Cu_9_S_5_ lattice creates available cationic sites for foreign cation incorporation into the vulnerable copper sulfide lattice thereby forming NaCuS-(M). NaCuS-(O) cannot be attributed to a crystallographic transformation and forms in the absence of the Cu_9_S_5_ phase. To confirm this, the copper precursor was added to a solution of Na-oleate at 320 °C followed by sulfur injection. The results yield Na_3_Cu_4_S_4_ formation without Cu_9_S_5_ forming first (Figure S13). The formation of NaCuS-(O) occurs by secondary nucleation rather than through the transformation of NaCuS-(M) into NaCuS-(O) as confirmed in Figure S14. We show the structures are not related or interchangeable, as after NaCuS-(O) formation, an injection of the highly reactive DDT did not induce any changes to NaCuS-(O). At 320 °C, NaCuS-(O), NaCuS-(M), and Cu_9_S_5_ are present in the reaction system. The line scan in Figure S10f shows a mixture of round and cuboid-shaped particles, and a different ratio of Na:Cu:S is observed in the line scan when comparing the round to cuboid particles. Formation of both Na–Cu–S NCs is described by the schematic depiction of phase evolution in Figure 5a (for additional details on the analysis, see section 6 of the SI).

**Figure 4:**
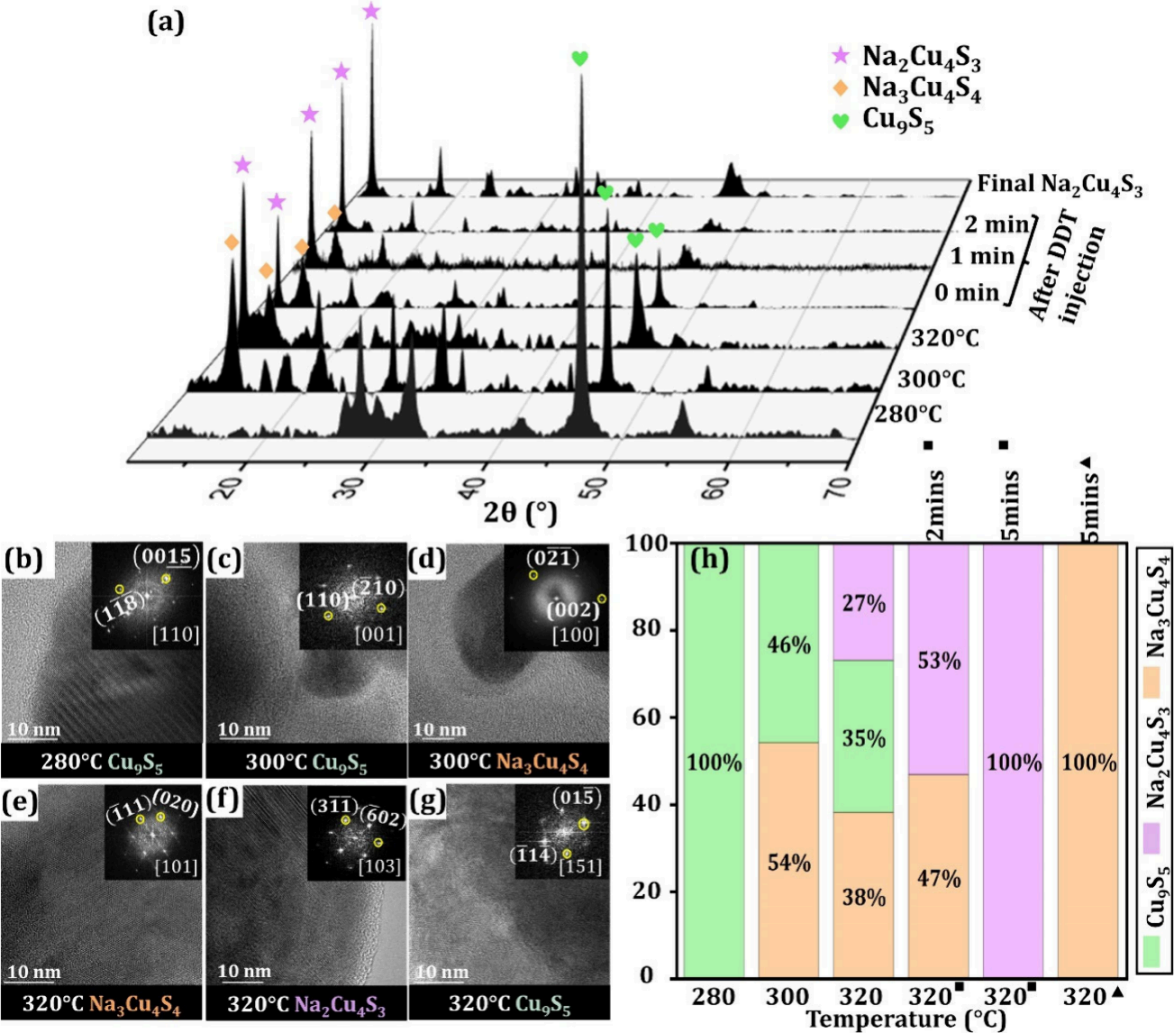
Aliquot study for the heat up and growth stages of NaCuS-(M) and NaCuS-(O) formation. (a) XRD patterns for heat up stage and NaCuS-(M) growth after DDT injection. (b–g) TEM and HRTEM of 280, 300, and 320 °C aliquots. (h) Phase percentages for the aliquots calculated from Rietveld refinement of the XRD patterns (■ = after DDT injection; ▲ = after TBDS injection).

**Figure 5:**
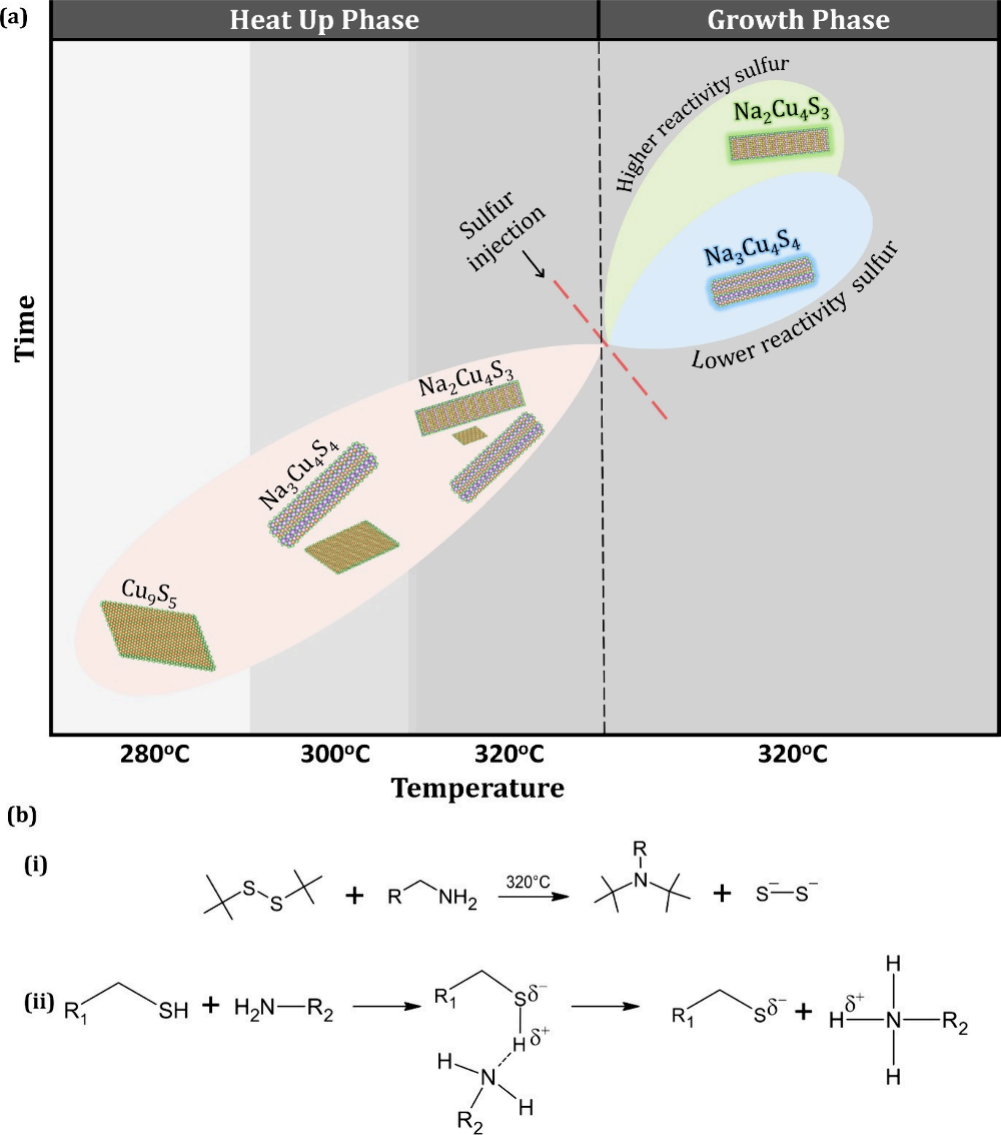
(a) Crystallographic transformation throughout the synthesis of Na_2_Cu_4_S_3_ and Na_3_Cu_4_S_4_. (b) Schematic depicting the precursor decomposition mechanisms for (i) TBDS and (ii) 1-DDT.

The growth of Na–Cu–S particles has a broad size distribution due to the nucleation and growth following the Finke–Watzky two-step mechanism whereby there is a slow continuous nucleation happening, and thus, we see shorter particles among the longer cuboids.^46^ In the formation of pure NaCuS-(O) and NaCuS-(M), the injection of a sulfur precursor at 320 °C significantly increased the concentration of sulfur species in the reaction system. This abrupt increase in sulfur availability plays a crucial role in indicating the reaction kinetics and subsequent NC formation. The time-dependent aliquot study shows that when TBDS was injected, thermodynamically stable NaCuS-(O) was formed swiftly, as evident from the XRD spectra (Figure S12) of aliquots taken after the injection. Contrary to this, when 1-DDT is injected, formation of the metastable NaCuS-(M) is observed. The selectivity of the phases by the variation of the S source could be explained by considering the C–S bond in these S sources. As investigated previously,^47−50^ TBDS, an organyl disulfide, displays ∼58 kcal mol^–1^ as the bond dissociation energy (BDE) for the C–S bond. At higher temperatures, it undergoes a sluggish species formation reaction to produce S_2_^2–^ species into the reaction system (Figure 5b(i)). This slow supply of S_2_^2–^ species then provides sufficient time to reorganize the crystalline phases toward the favorable production of thermodynamically stable NaCuS-(O). 1-DDT also displays the C–S bond dissociation energy in a similar range of 55 to 60 kcal mol^–1^. However, when OLA is present in the reaction mixture, 1-DDT tends to form a Lewis acid–base adduct (Figure 5b(ii)). This increases the activity of 1-DDT to produce a reactive thiolate species from which the S will readily form bonds with the metals in the system.^51^ Given a faster rate of reactive sulfide species, the crystal phase of the final product is arrested in kinetically metastable NaCuS-(M). To test this hypothesis further, in a separate set of experiments, we injected different S sources to form the Na–Cu–S NCs (Figures S15–S17). It was observed that S sources where the sulfur bonds are lower in energy and therefore higher in reactivity, e.g., 1-DDT and thiourea, favored the formation of NaCuS-(M). Contrary to this, S sources such as dipropyl disulfide with a comparatively higher C–S BDE of 65.2 kcal mol^–1^ result in NaCuS-(O) NCs.^52^ The conventional S source, S powder in the OLA, undergoes a transformation process involving the formation of radical anions S_n-2_^2–^ and alkane thioamide through S–S bond scission. However, this bond-breaking reaction proceeds slowly, resulting in a limited supply rate of S species in the reaction flask. This controlled release of S ultimately favors the formation of NaCuS-(O).

In conclusion, we report the first controlled synthesis of two stoichiometries with very distinct phase and electronic properties in the sustainable yet unexplored Na–Cu–S system: Na_3_Cu_4_S_4_ and Na_2_Cu_4_S_3_. Based on theoretical calculations, these phases are predicted to exhibit metallic (Na_3_Cu_4_S_4_) and semiconducting (Na_2_Cu_4_S_3_) behavior. To tackle the inherent difference in reactivity between sodium and copper precursors with sulfur, we designed a template-based synthesis approach. In this method, an in situ-formed cation-deficient Cu–S serves as a stable reaction intermediate, facilitating the incorporation of Na ions at high temperatures to produce the final Na–Cu–S NCs. Further, the phase and stoichiometry control in the Na–Cu–S system was achieved by modulating the excess sulfur supply in the reaction flask. The detailed mechanistic investigation combined with surface chemistry and electronic property analysis via experimental and computational studies demonstrates the ability to tune the bands precisely between two distinct band structures. This new understanding lays the groundwork for future research across a wide range of ABZ systems, potentially unlocking novel materials with tailored properties for diverse applications.

## Supporting Information Available

Na–Cu–S phase diagram; additional data of XRD, TEM, and XPS; additional data on band structures and absorption; analysis of the aliquot samples; control reactions; experimental details (PDF)

## References

[ref1] McKeever H.; Patil N. N.; Palabathuni M.; Singh S. Functional Alkali Metal-Based Ternary Chalcogenides: Design, Properties, and Opportunities. Chem. Mater. 2023, 35, 9833–9846. 10.1021/acs.chemmater.3c01652. 38107194 PMC10720346

[ref2] Mahmoud M. M. A.; Joubert D. P.; Molepo M. P. Structural, Stability and Thermoelectric Properties for the Monoclinic Phase of NaSbS_2_ and NaSbSe_2_: A Theoretical Investigation. Eur. Phys. J. B 2019, 92, 214 10.1140/epjb/e2019-90712-y.

[ref3] Ma N.; Jia F.; Xiong L.; Chen L.; Li Y.-Y.; Wu L.-M. CsCu_5_S_3_: Promising Thermoelectric Material with Enhanced Phase Transition Temperature. Inorg. Chem. 2019, 58, 1371–1376. 10.1021/acs.inorgchem.8b02919. 30620570

[ref4] Chen Y.; Shen Y.; Li X.; Sun J.; Wang Q. Β-CsCu_5_Se_3_: A Promising Thermoelectric Material Going beyond Photovoltaic Application. Adv. Theory Simul. 2020, 3, 2000169 10.1002/adts.202000169.

[ref5] Ma N.; Li Y.-Y.; Chen L.; Wu L.-M. α-CsCu_5_Se_3_: Discovery of a Low-Cost Bulk Selenide with High Thermoelectric Performance. J. Am. Chem. Soc. 2020, 142, 5293–5303. 10.1021/jacs.0c00062. 32118412

[ref6] Medina-Gonzalez A. M.; Yox P.; Chen Y.; Adamson M. A. S.; Rosales B. A.; Svay M.; Smith E. A.; Schaller R. D.; Wu K.; Rossini A. J.; Kovnir K.; Vela J. Solution-Grown Ternary Semiconductors: Nanostructuring and Stereoelectronic Lone Pair Distortions in I-V-VI2Materials. Chem. Mater. 2022, 34, 7357–7368. 10.1021/acs.chemmater.2c01410.

[ref7] Rosales B. A.; White M. A.; Vela J. Solution-Grown Sodium Bismuth Dichalcogenides: Toward Earth-Abundant, Biocompatible Semiconductors. J. Am. Chem. Soc. 2018, 140, 3736–3742. 10.1021/jacs.7b12873. 29451789

[ref8] Huang Y.-T.; Kavanagh S. R.; Righetto M.; Rusu M.; Levine I.; Unold T.; Zelewski S. J.; Sneyd A. J.; Zhang K.; Dai L.; Britton A. J.; Ye J.; Julin J.; Napari M.; Zhang Z.; Xiao J.; Laitinen M.; Torrente-Murciano L.; Stranks S. D.; Rao A.; Herz L. M.; Scanlon D. O.; Walsh A.; Hoye R. L. Z. Strong Absorption and Ultrafast Localisation in NaBiS_2_ Nanocrystals with Slow Charge-Carrier Recombination. Nat. Commun. 2022, 13, 4960 10.1038/s41467-022-32669-3. 36002464 PMC9402705

[ref9] Yang C.; Wang Z.; Lv Y.; Yuan R.; Wu Y.; Zhang W.-H. Colloidal CsCu_5_S_3_ Nanocrystals as an Interlayer in High-Performance Perovskite Solar Cells with an Efficiency of 22.29%. Chem. Eng. J. 2021, 406, 126855 10.1016/j.cej.2020.126855.

[ref10] Liu D.; Huang S.; Wang X.; Sa R. (Li,Na)SbS_2_ as a Promising Solar Absorber Material: A Theoretical Investigation. Spectrochim Acta A Mol. Biomol Spectrosc. 2021, 250, 119389 10.1016/j.saa.2020.119389. 33422871

[ref11] Parveen A.; Vaitheeswaran G. Exploring Exemplary Optoelectronic and Charge Transport Properties of KCuX(X = Se,Te). Sci. Rep. 2018, 8, 13071 10.1038/s41598-018-31300-0. 30166554 PMC6117315

[ref12] Peplinski Z.; Brown D. B.; Watt T.; Hatfield W. E.; Day P. Electrical Properties of Na_3_Cu_4_S_4_, a Mixed-Valence One-Dimensional Metal. Inorg. Chem. 1982, 21, 1752–1755. 10.1021/ic00135a010.

[ref13] Burschka C. Na_3_Cu_4_S_4_ - a Thiocuprate with Isolated 1∞[Cu_4_S_4_-Chains. Z. Nat. 1979, 34, 396–397. 10.1515/znb-1979-0308.

[ref14] Klepp K. O.; Sing M.; Boller H. Preparation and Crystal Structure of Na_7_Cu_12_S_10_, a Mixed Valent Thiocuprate with a Pseudo-One-Dimensional Structure. J. Alloys. Compd. 1993, 198, 25–30. 10.1016/0925-8388(93)90138-D.

[ref15] Bai C.; Wu Y.; Xin Y.; Mou J.; Xia L.; Ding D.; Zheng X.; Yu P. Alkali Metal Doped Copper-Sulfides as a New Class Electrocatalysts for Oxygen Evolution Reaction. J. Alloys. Compd. 2023, 962, 171171 10.1016/j.jallcom.2023.171171.

[ref16] Jain A.; Ong S. P.; Hautier G.; Chen W.; Richards W. D.; Dacek S.; Cholia S.; Gunter D.; Skinner D.; Ceder G.; Persson K. A. Commentary: The Materials Project: A Materials Genome Approach to Accelerating Materials Innovation. APL Mater. 2013, 1, 011002 10.1063/1.4812323.

[ref17] Yu Y.-X. Sodium/Potassium Intercalation on the Cu_4_S_4_ Nanosheet Accompanied by a Surface Phase Transition and Their Competition with Protons. ACS Appl. Energy Mater. 2023, 6, 10048–10060. 10.1021/acsaem.3c01623.

[ref18] Novikov S. A.; Casey J.; Long H. A.; Bledsoe J. C.; Locklin J. J.; Klepov V. V. Electron Deficiency in 2D Chalcogenide NaCu_4_S_3_ with Metallic Properties. Cryst. Growth Des. 2023, 23, 7243–7251. 10.1021/acs.cgd.3c00649.

[ref19] Ivanova T. M.; Maslakov K. I.; Sidorov A. A.; Kiskin M. A.; Linko R. V.; Savilov S. V.; Lunin V. V.; Eremenko I. L. XPS Detection of Unusual Cu(II) to Cu(I) Transition on the Surface of Complexes with Redox-Active Ligands. J. Electron Spectrosc. Relat. Phenom. 2020, 238, 146878 10.1016/j.elspec.2019.06.010.

[ref20] Roth A. N.; Chen Y.; Adamson M. A. S.; Gi E.; Wagner M.; Rossini A. J.; Vela J. Alkaline-Earth Chalcogenide Nanocrystals: Solution-Phase Synthesis, Surface Chemistry, and Stability. ACS Nano 2022, 16, 12024–12035. 10.1021/acsnano.2c02116. 35849721

[ref21] Lee S.; Sagar L. K.; Li X.; Dong Y.; Chen B.; Gao Y.; Ma D.; Levina L.; Grenville A.; Hoogland S.; García de Arquer F. P.; Sargent E. H. InP-Quantum-Dot-in-ZnS-Matrix Solids for Thermal and Air Stability. Chem. Mater. 2020, 32, 9584–9590. 10.1021/acs.chemmater.0c02870.

[ref22] Mphahlele L. L. R.; Ajibade P. A. Synthesis and Crystal Structure of Bis(Morpholino Dithiocarbamato) Cd(II) Complex and Its Use as Precursor for CdS Quantum Dots Using Different Capping Agents. J. Sulfur Chem. 2019, 40, 648–663. 10.1080/17415993.2019.1637876.

[ref23] Bai L.; Hu H.; Fu W.; Wan J.; Cheng X.; Zhuge L.; Xiong L.; Chen Q. Synthesis of a Novel Silica-Supported Dithiocarbamate Adsorbent and Its Properties for the Removal of Heavy Metal Ions. J. Hazard. Mater. 2011, 195, 261–275. 10.1016/j.jhazmat.2011.08.038. 21889843

[ref24] Kresse G.; Hafner J. Ab Initio Molecular Dynamics for Liquid Metals. Phys. Rev. B 1993, 47, 558–561. 10.1103/PhysRevB.47.558. 10004490

[ref25] Kresse G.; Furthmüller J. Efficient Iterative Schemes for Ab Initio Total-Energy Calculations Using a Plane-Wave Basis Set. Phys. Rev. B 1996, 54, 11169–11186. 10.1103/PhysRevB.54.11169. 9984901

[ref26] Kresse G.; Joubert D. From Ultrasoft Pseudopotentials to the Projector Augmented-Wave Method. Phys. Rev. B 1999, 59, 1758–1775. 10.1103/PhysRevB.59.1758.

[ref27] Heyd J.; Scuseria G. E.; Ernzerhof M. Hybrid Functionals Based on a Screened Coulomb Potential. J. Chem. Phys. 2003, 118, 8207–8215. 10.1063/1.1564060.

[ref28] Krukau A. V.; Vydrov O. A.; Izmaylov A. F.; Scuseria G. E. Influence of the Exchange Screening Parameter on the Performance of Screened Hybrid Functionals. J. Chem. Phys. 2006, 125, 224106 10.1063/1.2404663. 17176133

[ref29] Perdew J. P.; Burke K.; Wang Y. Generalized Gradient Approximation for the Exchange-Correlation Hole of a Many-Electron System. Phys. Rev. B 1996, 54, 16533–16539. 10.1103/PhysRevB.54.16533. 9985776

[ref30] Yu L.; Zunger A. Identification of Potential Photovoltaic Absorbers Based on First-Principles Spectroscopic Screening of Materials. Phys. Rev. Lett. 2012, 108, 068701 10.1103/PhysRevLett.108.068701. 22401127

[ref31] Deringer V. L.; Tchougréeff A. L.; Dronskowski R. Crystal Orbital Hamilton Population (COHP) Analysis As Projected from Plane-Wave Basis Sets. J. Phys. Chem. A 2011, 115, 5461–5466. 10.1021/jp202489s. 21548594

[ref32] M Ganose A.; J Jackson A.; O Scanlon D. Sumo: Command-Line Tools for Plotting and Analysis of Periodic Ab Initio Calculations. J. Open Source Softw. 2018, 3, 717 10.21105/joss.00717.

[ref33] Savory C. N.; Ganose A. M.; Travis W.; Atri R. S.; Palgrave R. G.; Scanlon D. O. An Assessment of Silver Copper Sulfides for Photovoltaic Applications: Theoretical and Experimental Insights. J. Mater. Chem. A 2016, 4, 12648–12657. 10.1039/C6TA03376H. PMC5059790 27774149

[ref34] Wang Y.; Kavanagh S. R.; Burgués-Ceballos I.; Walsh A.; Scanlon D. O.; Konstantatos G. Cation Disorder Engineering Yields AgBiS_2_ Nanocrystals with Enhanced Optical Absorption for Efficient Ultrathin Solar Cells. Nat. Photonics 2022, 16, 235–241. 10.1038/s41566-021-00950-4.

[ref35] Dou W.; Spooner K. B.; Kavanagh S. R.; Zhou M.; Scanlon D. O. Band Degeneracy and Anisotropy Enhances Thermoelectric Performance from Sb_2_Si_2_Te_6_ to Sc_2_Si_2_Te_6_. J. Am. Chem. Soc. 2024, 146, 17679–17690. 10.1021/jacs.4c01838. 38889404 PMC11228999

[ref36] Gibbs Z. M.; Ricci F.; Li G.; Zhu H.; Persson K.; Ceder G.; Hautier G.; Jain A.; Snyder G. J. Effective Mass and Fermi Surface Complexity Factor from Ab Initio Band Structure Calculations. NPJ. Comput. Mater. 2017, 3, 8 10.1038/s41524-017-0013-3.

[ref37] Park J.; Xia Y.; Ozoliņš V.; Jain A. Optimal Band Structure for Thermoelectrics with Realistic Scattering and Bands. NPJ. Comput. Mater. 2021, 7, 43 10.1038/s41524-021-00512-w.

[ref38] Jung Y. K.; Kim J. Il; Lee J.-K. Thermal Decomposition Mechanism of Single-Molecule Precursors Forming Metal Sulfide Nanoparticles. J. Am. Chem. Soc. 2010, 132, 178–184. 10.1021/ja905353a. 20000670

[ref39] Liu Y.; Liu M.; Swihart M. T. Shape Evolution of Biconcave Djurleite Cu_1.94_S Nanoplatelets Produced from CuInS_2_ Nanoplatelets by Cation Exchange. J. Am. Chem. Soc. 2017, 139, 18598–18606. 10.1021/jacs.7b09577. 29200274

[ref40] Young H. L.; McCormick C. R.; Butterfield A. G.; Gomez E. D.; Schaak R. E. Postsynthetic Thiol-Induced Reshaping of Copper Sulfide Nanoparticles. Chem. Mater. 2022, 34, 11014–11025. 10.1021/acs.chemmater.2c03049.

[ref41] Shah I. D.; Khalafalla S. E. Kinetics and Mechanism of the Conversion of Covellite (CuS) to Digenite (Cu1.8S). Metall Trans 1971, 2, 2637–2643. 10.1007/BF02814907.

[ref42] Roseboom E. H. An Investigation of the System Cu-S and Some Natural Copper Sulfides between 25 Degrees and 700 Degrees C. Econ Geol 1966, 61, 641–672. 10.2113/gsecongeo.61.4.641.

[ref43] Sarapajevaite G.; Baltakys K. Thermal Stability and Decomposition Mechanism of Synthetic Covellite Samples. J. Therm. Anal. Calorim. 2022, 147, 10951–10963. 10.1007/s10973-022-11313-8.

[ref44] De Trizio L.; Manna L. Forging Colloidal Nanostructures via Cation Exchange Reactions. Chem. Rev. 2016, 116, 10852–10887. 10.1021/acs.chemrev.5b00739. 26891471 PMC5043423

[ref45] Steimle B. C.; Lord R. W.; Schaak R. E. Phosphine-Induced Phase Transition in Copper Sulfide Nanoparticles Prior to Initiation of a Cation Exchange Reaction. J. Am. Chem. Soc. 2020, 142, 13345–13349. 10.1021/jacs.0c06602. 32700901

[ref46] Watzky M. A.; Finke R. G. Transition Metal Nanocluster Formation Kinetic and Mechanistic Studies. A New Mechanism When Hydrogen Is the Reductant: Slow, Continuous Nucleation and Fast Autocatalytic Surface Growth. J. Am. Chem. Soc. 1997, 119, 10382–10400. 10.1021/ja9705102.

[ref47] Kapuria N.; Patil N. N.; Sankaran A.; Laffir F.; Geaney H.; Magner E.; Scanlon M.; Ryan K. M.; Singh S. Engineering Polymorphs in Colloidal Metal Dichalcogenides: Precursor-Mediated Phase Control, Molecular Insights into Crystallisation Kinetics and Promising Electrochemical Activity. J. Mater. Chem. A 2023, 11, 11341–11353. 10.1039/D2TA09892J.

[ref48] Rhodes J. M.; Jones C. A.; Thal L. B.; Macdonald J. E. Phase-Controlled Colloidal Syntheses of Iron Sulfide Nanocrystals via Sulfur Precursor Reactivity and Direct Pyrite Precipitation. Chem. Mater. 2017, 29, 8521–8530. 10.1021/acs.chemmater.7b03550.

[ref49] Guo Y.; Alvarado S. R.; Barclay J. D.; Vela J. Shape-Programmed Nanofabrication: Understanding the Reactivity of Dichalcogenide Precursors. ACS Nano 2013, 7, 3616–3626. 10.1021/nn400596e. 23517277

[ref50] Brutchey R. L. Diorganyl Dichalcogenides as Useful Synthons for Colloidal Semiconductor Nanocrystals. Acc. Chem. Res. 2015, 48, 2918–2926. 10.1021/acs.accounts.5b00362. 26545235

[ref51] Koskela K. M.; Strumolo M. J.; Brutchey R. L. Progress of Thiol-Amine ‘Alkahest’ Solutions for Thin Film Deposition. Trends Chem. 2021, 3, 1061–1073. 10.1016/j.trechm.2021.09.006.

[ref52] Yang Y.; Yu H.; Sun X.; Dang Z. Density Functional Theory Calculations on S―S Bond Dissociation Energies of Disulfides. J. Phys. Org. Chem. 2016, 29, 6–13. 10.1002/poc.3480.

