# Supplementary material for: Ligand-Assisted Colloidal Synthesis of Alkali Metal-Based Ternary Chalcogenide: Nanostructuring and Phase Control in Na–Cu–S System
Source: Nano Lett. 2025 Mar 12;25(12):4652–8. doi: 10.1021/acs.nanolett.4c04257 (PMC11951143; doi:10.1021/acs.nanolett.4c04257)
Supplement: Supplementary file 1 — nl4c04257_si_001.pdf [file nl4c04257_si_001.pdf]

Ligand Assisted Colloidal Synthesis of Alkali Metal-based Ternary Chalcogenide: Nanostructuring and Phase Control in Na-Cu-S System

*Hannah McKeever[1], Nilotpal Kapuria [1,2], Adair Nicolson [3,4], Suvodeep Sen [1], David Scanlon [3], Kevin M Ryan[1], Shalini Singh[1]**

[1] Department of Chemical Sciences and Bernal Institute, University of Limerick, Limerick, Ireland, V94 T9PX

[2] Department of Chemistry, Indiana University – Bloomington, 800 East Kirkwood Avenue, Bloomington, Indiana

[3] School of Chemistry, University of Birmingham, Birmingham, United Kingdom,

B15 2TT

[4] Department of Chemistry, University College London, London, United Kingdom,

WC1H 0AJ

Contents

1. Material Properties 3

2. Experimental 3

2.1 Materials 3

2.2 Synthesis of copper diethyldithiocarbamate 4

2.3 Synthesis of $Na_3Cu_4S_4$ 4

2.4 Synthesis of $Na_2Cu_4S_3$ 5

2.6 General Safety Handling 6

2.7 Aliquot Study 7

3. Additional Characterisation 7
3.1 XRD analysis of $Na_3Cu_4S_4$ 7
3.2 XRD analysis of $Na_2Cu_4S_3$ 8
3.3 TEM image of $Na_3Cu_4S_4$ for size distribution analysis 9
3.4 TEM image of $Na_2Cu_4S_3$ for size distribution analysis 9
3.5 STEM-EDS elemental maps for $Na_3Cu_4S_4$ 10
3.6 STEM-EDS elemental maps for $Na_2Cu_4S_3$ 10
4.0 X-ray Photoelectron Spectroscopy (XPS) 11
4.1 Full XPS Spectra 11
5.0 Band structure 12
5.1 Band structure of $Na_3Cu_4S_3$ 12
5.2 DFT calculated Tauc plot for $Na_2Cu_4S_3$ 12
6. Aliquot Study 13
6.1 Low resolution TEM and STEM-EDS line scans of aliquots 15
6.2 XRD of Aliquot taken at 170°C 16
6.3 XRD analysis of time dependent aliquot study 16
7. Control Reactions 17
7.1 XRD of control for Cu9S5 transformation 17
7.2 XRD of control to show $Na_3Cu_4S_4$ does not transform into $Na_2Cu_4S_3$ 18
7.3 XRD analysis of control experiments for $Na_2Cu_4S_3$ 19
7.4 XRD analysis of control experiments for $Na_3Cu_4S_4$ 20
7.5 TEM images of control experiments for $Na_3Cu_4S_4$ 20
8. Analysis Methods 21
8.1 X-ray Diffraction (XRD) 21
8.2 Rietveld Refinement 21
8.3 Electron Microscopy 21
8.4 X-ray Photoelectron Spectroscopy (XPS) 21
8.5 Fourier Transform Infrared Spectroscopy 22
8.6 Computational Methods 22

1. Material Properties

	$Na_3Cu_4S_4$	$Na_2Cu_4S_3$
**Acronym**	**NaCuS-(O)**	**NaCuS-(M)**
**Crystal Structure**	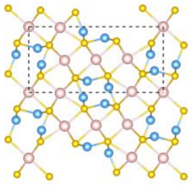	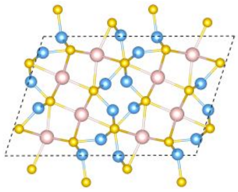
**Crystal system**	**Orthorhombic**	**Monoclinic**
**Electronic Nature**	**Metallic**	**Semi Conducting**
**Band gap**	**N/A**	**1.6eV**
**Sulfur Precursor Reactivity**	**Low**	**High**

Table S1 Characteristics and properties of $Na_3Cu_4S_4$ and $Na_2Cu_4S_3$.

2. Experimental

2.1 Materials

Sodium oleate purchased from TCI, 1-dodecanethiol (1-DDT),≥98%, oleylamine(OLA, 70%), Di-tert-butyl disulfide (TBDS), Dipropyl disulfide 99%, Thiourea, Tetra ethylene glycol dimethyl ether (TEGDME), Sulfur, purchased from Sigma-Aldrich. Toluene (Tol), Isopropanol (IPA) were purchased from Honeywell.

The chemicals were used as received without any further purification.

2.2 Synthesis of copper diethyldithiocarbamate

For the synthesis of copper diethyldithiocarbamate, 9.0 g of sodium diethyldithiocarbamate was dissolved in 150 mL of deionised water. 4.23 g of copper chloride was dissolved in 50 mL of deionised water. The solution of copper chloride was stirred constantly and the sodium diethyldithiocarbamate was added dropwise to the copper chloride solution. All reactions took place at room temperature. A black precipitate formed. This black precipitate was washed with water by a Büchner funnel until the solution in the funnel ran colourless. The precipitate was dried overnight under vacuum at 80 °C. After 24 hours the black copper diethyldithiocarbamate underwent recrystallisation for further purification. Chloroform was heated to approximately 60 °C and the copper diethyldithiocarbamate crystals were dissolved in it. The solution was allowed to cool to room temperature to allow for crystallisation and growth. The crystals were then filtered out using vacuum filtration with a buchner funnel and the resulting crystals were dried overnight under vacuum at 80 °C.

2.3 Synthesis of $Na_3Cu_4S_4$

In a synthesis of $Na_3Cu_4S_4$, 60.9 mg (0.2 mmol) Sodium Oleate and 57.6 mg (0.16 mmol) Cu-DDTC were weighed out into a three neck round bottomed flask (RBF). 10 mL of OLA was added to the flask as a solvent. The reaction mixture was put under vacuum and the temperature was ramped for 5 minutes to 120 °C. The mixture was evacuated at 120 °C for 60 minutes with a vacuum pressure > 200 mTorr. When the 60 minutes had elapsed the reaction mixture was exposed to an argon atmosphere and ramped to 320 °C (15 minutes ramp). When the temperature reached 320 °C, 0.8 mL of TBDS (4 mmol) was rapidly injected into the reaction mixture. After the injection the temperature was allowed to recover to 320 °C (t=0) before the reaction time of 5 minutes proceeded. At t=5 minutes the heating mantle was removed to terminate the reaction, when the temperature dropped to 250 °C, a cold-water bath was placed around the vessel to rapidly cool the reaction mixture. When the temperature reached 80 °C,

10 mL of toluene was injected into the flask. The reaction mixture was then transferred to a 50 mL centrifuge tube and was mixed by vortexing. Next, 10 mL IPA was added to the mixture and vortexed, this was followed by 10 minutes of sonication and subsequently 5 minutes of centrifugation at 5000 RPM. The supernatant was discarded, and the pellet underwent a further 3 washes. These washes followed the same procedure, as follows: addition of 5 mL toluene, mixing by vertexing, addition of 5 mL IPA, mixing by vertexing, 10-minute sonication, 5 minutes of centrifugation at 5000 RPM. Sample was stored in a small volume of toluene.

2.4 Synthesis of $Na_2Cu_4S_3$

In a synthesis of $Na_2Cu_4S_3$, 60.9 mg (0.2 mmol) Sodium Oleate and 57.6mg (0.16 mmol) Cu-DDTC were weighed out into a three neck round bottomed flask (RBF). 10 mL of OLA was added to the flask as a solvent. The reaction mixture was put under vacuum and the temperature was ramped for 5 minutes to 120 °C. The mixture was evacuated at 120 °C for 60 minutes with a vacuum pressure > 200 mTorr. When the 60 minutes had elapsed the reaction mixture was exposed to an argon atmosphere and ramped to 320 °C (15 minutes ramp). When the temperature reached 320 °C, 2mL of DDT (8mmol) in 4ml OLA was rapidly injected into the reaction mixture. After the injection the temperature was allowed to recover to 320 °C (t=0) before the reaction time of 5 minutes proceeded. At t=5 minutes the heating mantle was removed to terminate the reaction, when the temperature dropped to 250 °C, a cold-water bath was placed around the vessel to rapidly cool the reaction mixture. When the temperature reached 80 °C, 10 mL of toluene was injected into the flask. The reaction mixture was then transferred to a 50 mL centrifuge tube and was mixed by vortexing. Next, 10 mL IPA was added to the mixture and vortexed, this was followed by 10 minutes of sonication and subsequently 5 minutes of centrifugation at 5000 RPM. The supernatant was discarded, and the pellet underwent a further 3 washes. These washes followed the same procedure, as follows: addition of 5mL toluene, mixing by vertexing, addition of 5 mL IPA, mixing by vertexing, 10-

minute sonication, 5 minutes of centrifugation at 5000 RPM. Sample was stored in a small volume of toluene.

2.5 Synthesis of $NaCu_5S_3$ and control experiments

A molarity of 8 mmol was used for the sulfur precursors in the experiments. For precursors with 2 sulfur atoms (TBDS and dipropyl disulfide) a molarity of 4mmol was used to maintain the same molar ratio. For the synthesis of $NaCu_5S_{3,}$ 8mmol of Thiourea was dissolved in 8ml of OLA and used as the sulfur injection.

2.6 General Safety Handling

Personal protective equipment (PPE) (lab coat, gloves and lab glasses) should be worn at all times. Hazard identification should be carried out and considered for all chemicals in use using safety data sheet's (SDS's) from the supplier. Training in the use of a Schlenk line with vacuum and argon atmosphere is essential before carrying out a synthesis. A nitrogen trap should be filled with liquid nitrogen before use of the Schlenk line under vacuum to condense any gases produced during the reaction. High temperatures are employed during the reaction and care must be taken when handling the heating mantle at the end of the reaction, ensure it is positioned safely once removed from the reaction vessel. The entire synthesis, including the weighing and measuring of chemicals and the washing of nanoparticles should be carried out in a fumehood. Oleylamine is corrosive and a health hazard and should be handled with extreme care using PPE in a fumehood. 1-DDT is corrosive, has moderate health hazards and has a strong odour and should be handled with care. Contaminated gloves or tissues should be sealed in a polyethylene bag in a fumehood and then disposed of. When using needles for injections, care should be taken, and the one hand scoop method should be used for re-sheathing. Care was taken during the aliquot study to not directly hold the glass syringe when removing high temperature aliquots.

2.7 Aliquot Study

During the reaction 5 mL portions of the reaction solution were removed from the RBF using a glass syringe and immediately quenched in 10 ml of toluene in a glass vial. This method was used to avoid effects to the concentration of reactants in the solution and to ensure immediate quenching of the reaction to gain the most accurate results for each time point. Once cooled to room temperature the contents of the glass vial were transferred to an Eppendorf tube and mixed by vortexing. Next, 10 mL IPA was added to the mixture in the Eppendorf tube and vortexed, this was followed by 10 minutes of sonication and subsequently 5 minutes of centrifugation at 5000 RPM. The supernatant was discarded, and the pellet underwent a further 3 washes using the same washing procedure mentioned for $Na_3Cu_4S_4$ and $Na_2Cu_4S_3$ nanocrystals.

3. Additional Characterisation

3.1 XRD analysis of $Na_3Cu_4S_4$

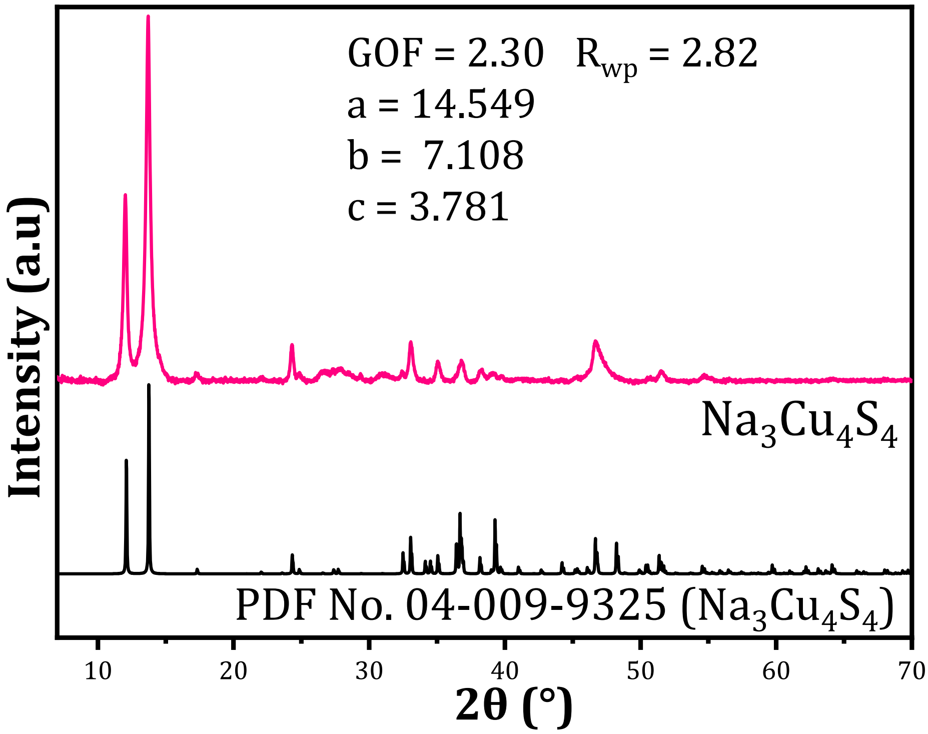

Figure S1 XRD analysis of $Na_3Cu_4S_4$ using Rietveld refinement, reference pattern PDF No. 04-009-9325.

The goodness of fit (GOF) and cell lengths were measured using Rietveld refinement. The cell lengths are in close agreement with the expected values of 14.62Å, 7.16Å and 3.77Å for a, b and c respectively.

3.2 XRD analysis of $Na_2Cu_4S_3$

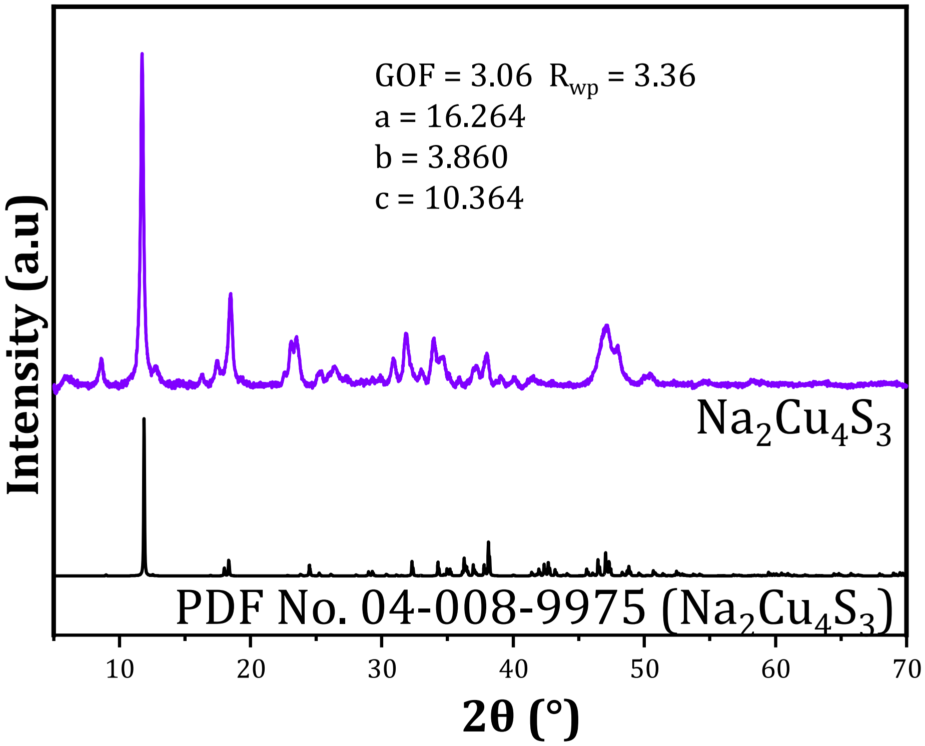

Figure S2 XRD analysis of $Na_2Cu_4S_3$, reference pattern PDF No. 04-088-9975.

Cell lengths calculated using Rietveld refinement of a= 16.264Å, b= 3.860Å, c= 10.364Å were observed, showing a good match to the expected values of a=15.63Å, b=3.86Å and c=10.33Å.

3.3 TEM image of $Na_3Cu_4S_4$ for size distribution analysis

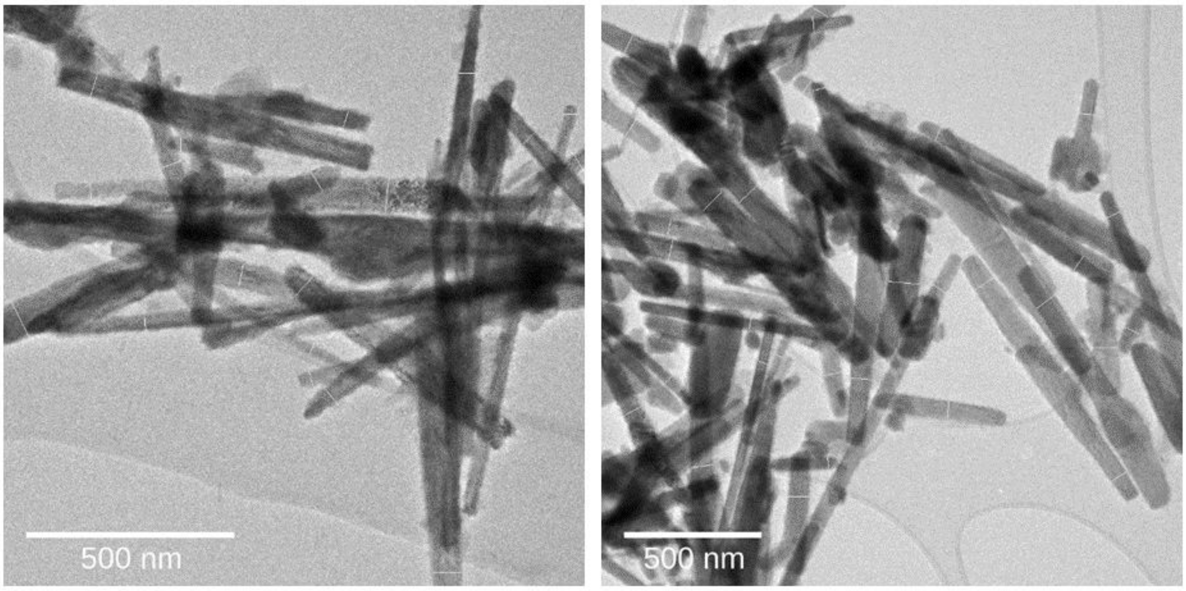

Figure S3 Low resolution TEM Images used for size distribution measurements of $Na_3Cu_4S_4$ (sample size 50 particles)

Due to the particles being nanoscale in only one direction it was difficult to obtain images with enough particles to make an accurate measurement of size distribution.

3.4 TEM image of $Na_2Cu_4S_3$ for size distribution analysis

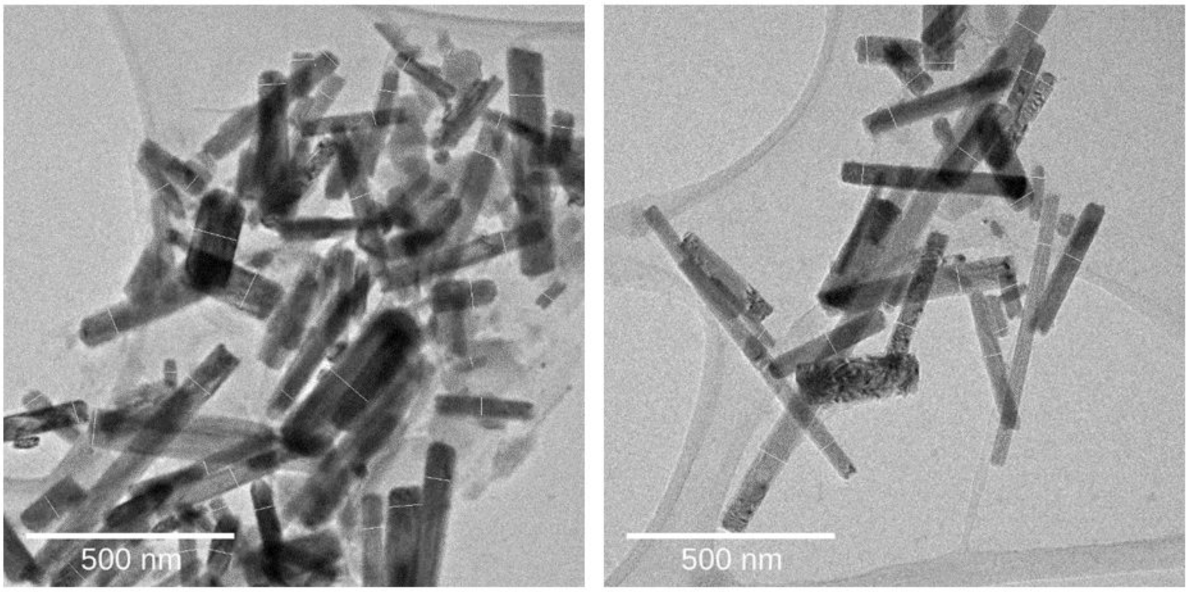

Figure S4 Low resolution TEM Images used for size distribution measurements of $Na_2Cu_4S_3$ (sample size 66 particles). Due to the particles being nanoscale in only one direction it was difficult to obtain images with enough particles to make an accurate measurement of size distribution.

3.5 STEM-EDS elemental maps for $Na_3Cu_4S_4$

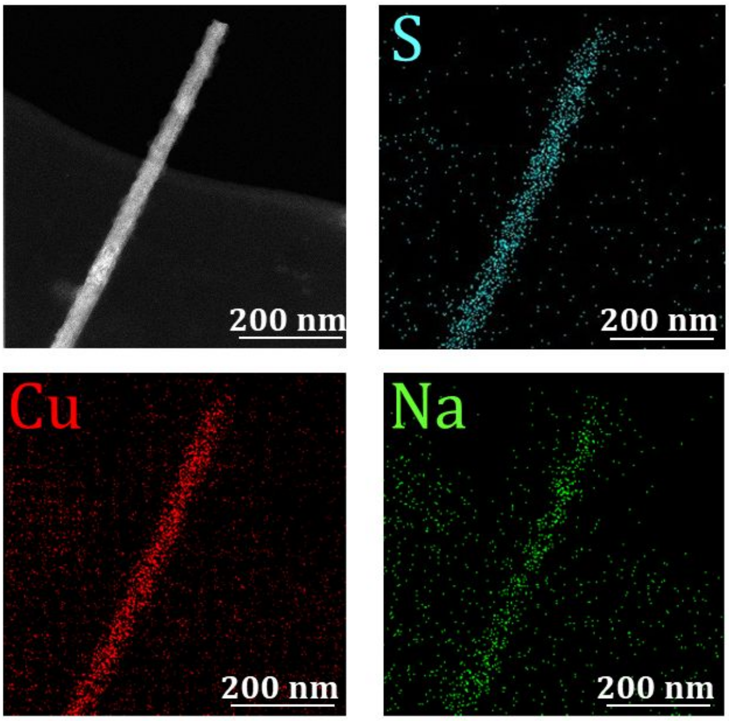

Figure S5 STEM-EDS elemental maps for S, Cu and Na for $Na_3Cu_4S_4$

3.6 STEM-EDS elemental maps for $Na_2Cu_4S_3$

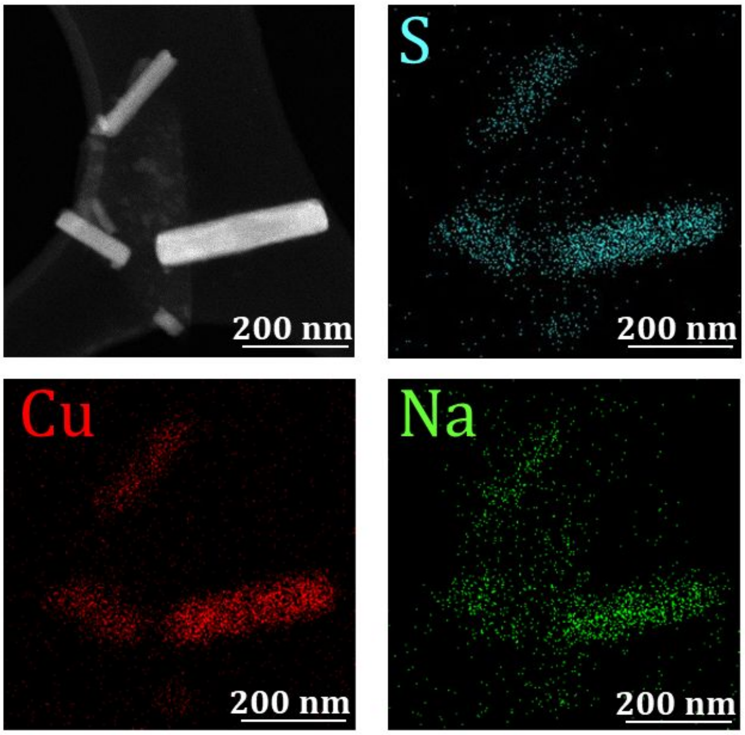

Figure S6 STEM-EDS elemental maps for S, Cu and Na for $Na_2Cu_4S_3$

4.0 X-ray Photoelectron Spectroscopy (XPS)

4.1 Full XPS Spectra

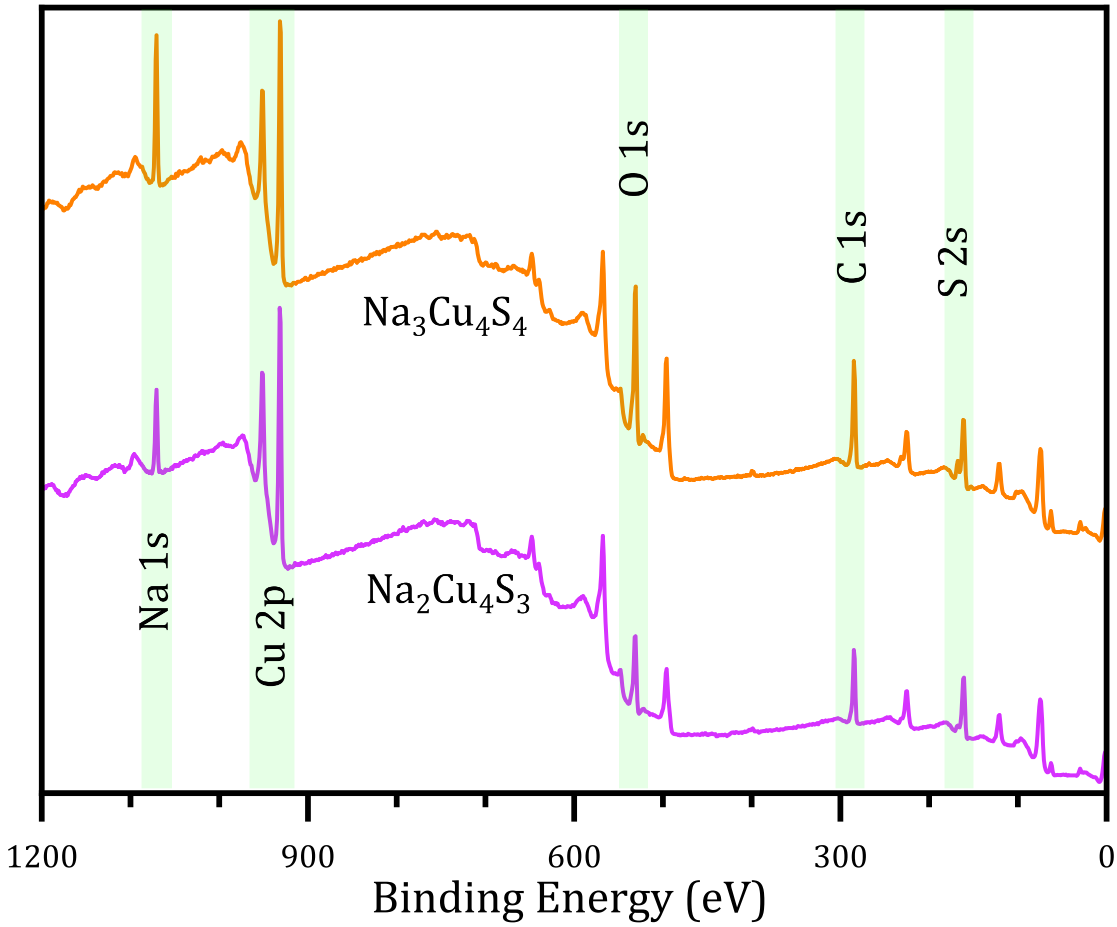

Figure S7 Full X-ray photoelectron spectroscopy (XPS) spectra for $Na_3Cu_4S_4$ and $Na_2Cu_4S_3$

5.0 Band structure

5.1 Band structure of $Na_3Cu_4S_3$

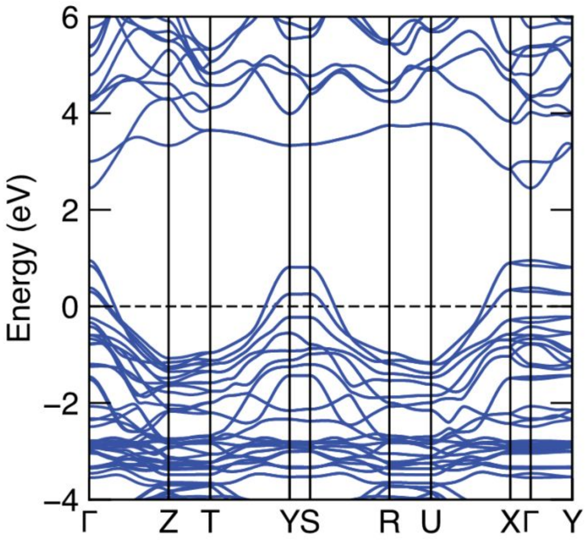

Figure S8 Electronic band structure of $Na_3Cu_4S_4$ calculated using HSE06

5.2 DFT calculated Tauc plot for $Na_2Cu_4S_3$

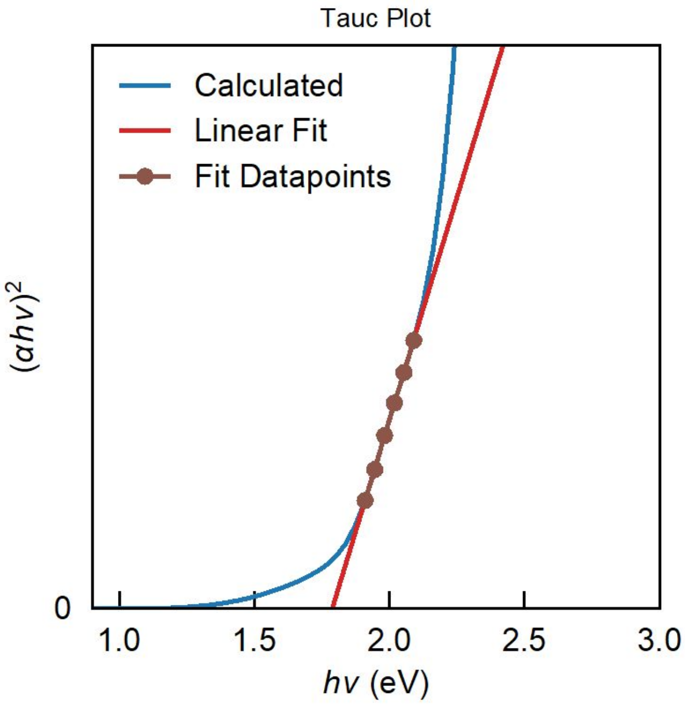

Figure S9 DFT calculated tauc plot giving a band gap of 1.79 eV for $Na_2Cu_4S_3$.

6. Aliquot Study

The reaction starts with the formation of copper sulfide at ~170°C. The XRD analysis of the aliquot withdrawn at 170°C confirms this as the XRD pattern displays peaks characteristic of hexagonal CuS (PDF No. 00-006-0464) (Figure S1.14). We do not expect to see sodium incorporation here based on Pearson's hard-soft acid-base theory. Na^+ is a hard Lewis acid while Cu^+ is a soft Lewis base and S^{2-} is a soft Lewis base. The soft acid-base pair has strong interactions causing the favourable formation of copper sulfide upon the decomposition of the dithiocarbamate precursor.[20,38] The lack of peaks associated with sodium compounds in the XRD confirms sodium remains in solution and does not participate in this initial nucleation. As the temperature is increased to 280°C Cu_9S_5 is observed, CuS transforms into Cu_9S_5 in the 170°C to 280°C temperature range. XRD (figure 4a) confirms the presence of trigonal Cu_9S_5 (PDF No. 00-047-1748) and CuS XRD peaks are no longer observed. The Cu_9S_5 crystal phase is further confirmed by HRTEM in figure 4b with d-spacing values of 3.3Å and 3.1Å for $(1\bar{8}\bar{8})$ and $(00\underline{15})$ planes matching with Cu_9S_5. This visualization of the particles from low resolution TEM (figure S1.13a) show their round shape and STEM-EDS line mapping in figure S1.13d confirms the presence of both copper and sulfur. No sodium signals are displayed in the STEM-EDS, confirming that it has yet to be incorporated in the crystalline samples and still remains in solution. As the temperature continues to ramp, we see sodium incorporation occurring with $Na_3Cu_4S_4$ forming at ~300°C. At this stage, this reaction mixture still contains particles of Cu_9S_5. The XRD in figure 4a shows a low intensity peak at 13.9° in the aliquot taken from when the reaction reached 300°C. This peak is characteristic of $Na_3Cu_4S_4$ (PDF No. 04-009-9325), confirming its formation. The Cu_9S_5 peak at 46.6° is also still present, indicating that both $Na_3Cu_4S_4$ and Cu_9S_5 are present in the reaction solution at this point. Low resolution TEM in figure S1.13b shows a broad distribution of shapes and sizes in the particles, where presence

of round particles and the beginning of elongation in some particles can be perceived. HRTEM corroborates the presence of both Cu_9S_5 and $Na_3Cu_4S_4$ (figure 4c & d) where Cu_9S_5 planes of ($\bar{2}$ 10) and (110) have d-spacings of ~4.9Å and ~5.0Å matching the reference pattern. The d-spacings of ~3.6Å and ~4.9Å for $Na_3Cu_4S_4$ planes ($0\bar{2}\bar{1}$) and (002) also match the reference data. The STEM-EDS line scan in figure S1.13e shows particles with the presence of Na, displaying its incorporation in the crystals. A further increase in temperature to 320°C presents the formation of $Na_2Cu_4S_3$ phase alongside $Na_3Cu_4S_4$ (with some Cu_9S_5 still remaining in the reaction solution). The most intense peak at 11.8° in the XRD spectra in figure 4a for the 320°C aliquot matches with $Na_2Cu_4S_3$ (PDF No. 04-088-9975). The main peak of $Na_3Cu_4S_4$ remains at a low intensity and the main Cu_9S_5 peak also remains present. HRTEM in figure 4e-g corroborates the presence of the three species, HRTEM d-spacings of each species was matched with the corresponding d-spacings in the reference patterns; $Na_3Cu_4S_4$ (planes ($\bar{1}11$) and (020) match with d-spacings 3.0Å and 2.9Å), $Na_2Cu_4S_3$ (planes ($3\bar{1}\bar{1}$) and ($\bar{6}02$) with d-spacings 3.2Å and 4.0Å) and Cu_9S_5 (planes ($\bar{1}1\bar{4}$) and ($01\bar{5}$) with d-spacings 3.1Å and 3.3Å). Low resolution TEM (figure S1.13c) shows a mixture of cuboid and spherical particles of varying sizes. When the temperature reaches 320°C, a sulfur precursor is injected and after 5 minutes of growth time a pure phase of $Na_3Cu_4S_4$ or $Na_2Cu_4S_3$ is observed, the stoichiometry depending on the sulfur precursor used. After the injection a time dependent aliquot study was carried out for both $Na_2Cu_4S_3$ and $Na_3Cu_4S_4$ (figure 4a and S1.15) with t=0 mins being the time at which the temperature recovers to 320°C after the sulfur precursor injection. The XRD spectra in figure 4a and S1.15 show that before an injection of either precursor the particles in solution are a mixture of Cu_9S_5, $Na_3Cu_4S_4$ and $Na_2Cu_4S_3$. When a swift injection of TBDS is added to the system, the formation of $Na_3Cu_4S_4$ occurs rapidly and the XRD spectra shows at T=0 mins the peaks are matching with the reference pattern of $Na_3Cu_4S_4$ giving a pure phase (figure S1.15). In the case of a swift injection of 1-DDT, it takes 5 minutes for the system to

form pure $Na_2Cu_4S_3$. The evolution of $Na_2Cu_4S_3$ formation can be seen through the XRD patterns (figure 4a) where 1- and 2-minute aliquots were taken. The main peak of orthorhombic $Na_3Cu_4S_4$ at 13.7° can be observed at a low intensity at the 1-minute timepoint and after 2 minutes the peak is still present, but the intensity is seen to decrease. By 5 minutes the peaks of $Na_3Cu_4S_4$ are no longer visible and a pure phase of monoclinic $Na_2Cu_4S_3$ is formed.

6.1 Low resolution TEM and STEM-EDS line scans of aliquots

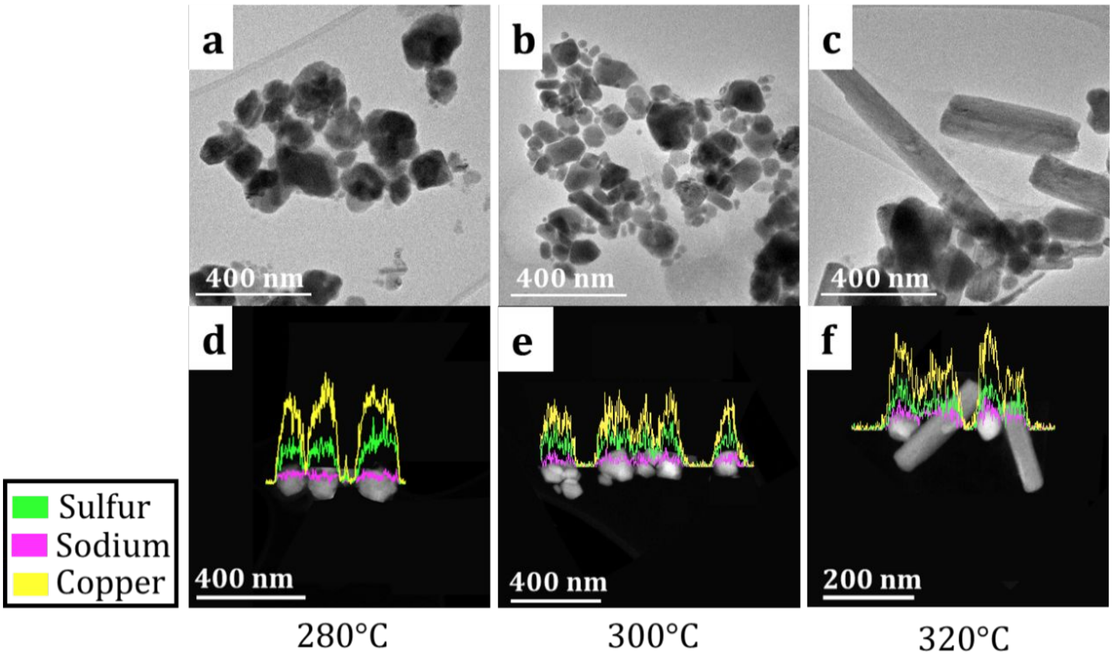

Figure S10 Low resolution TEM (a-c) and STEM-EDS line scans (d-f) of aliquots 280°C, 300 °C and 320 °C.

6.2 XRD of Aliquot taken at 170°C

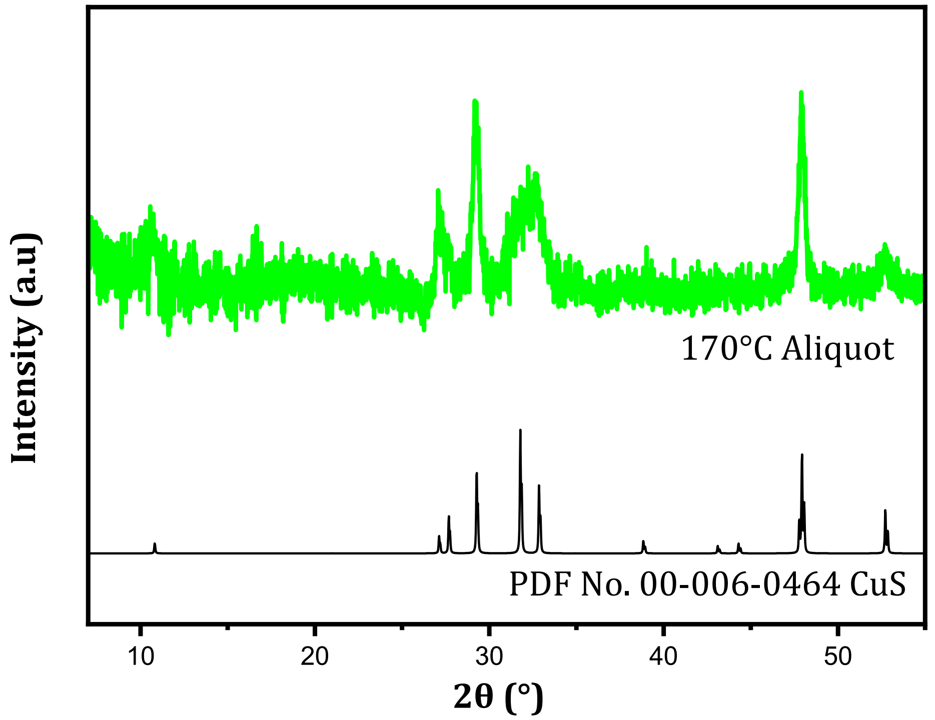

Figure S11 XRD Spectra of the Aliquot taken at 170°C with CuS reference pattern

6.3 XRD analysis of time dependent aliquot study

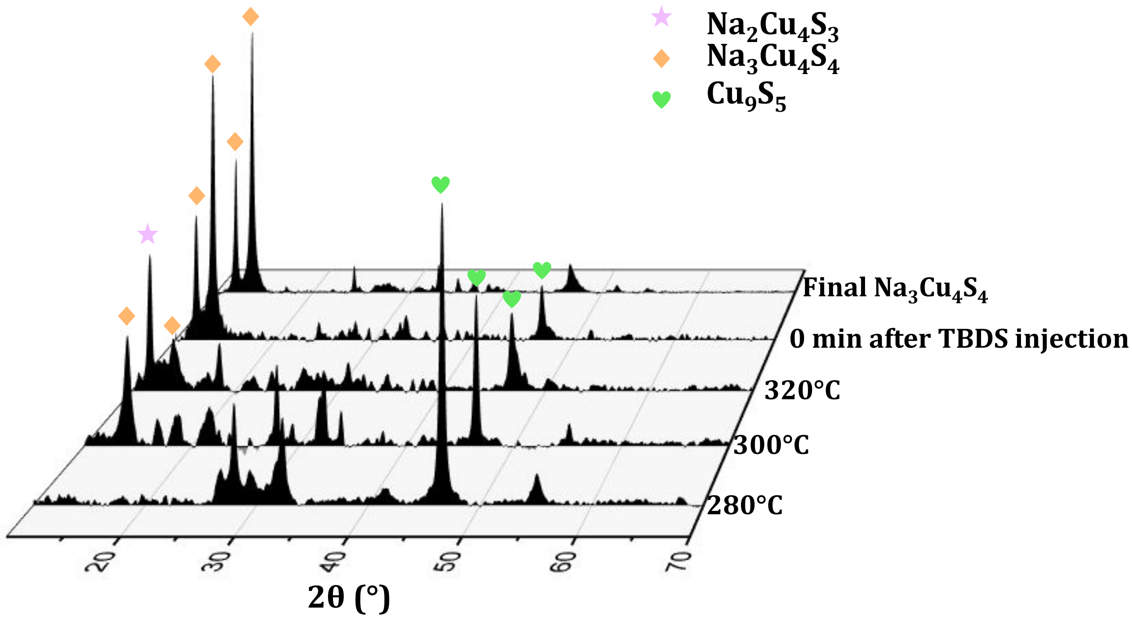

Figure S12 Aliquot study for the heat up and growth stages of $Na_3Cu_4S_4$ formation

7. Control Reactions

7.1 XRD of control for Cu9S5 transformation

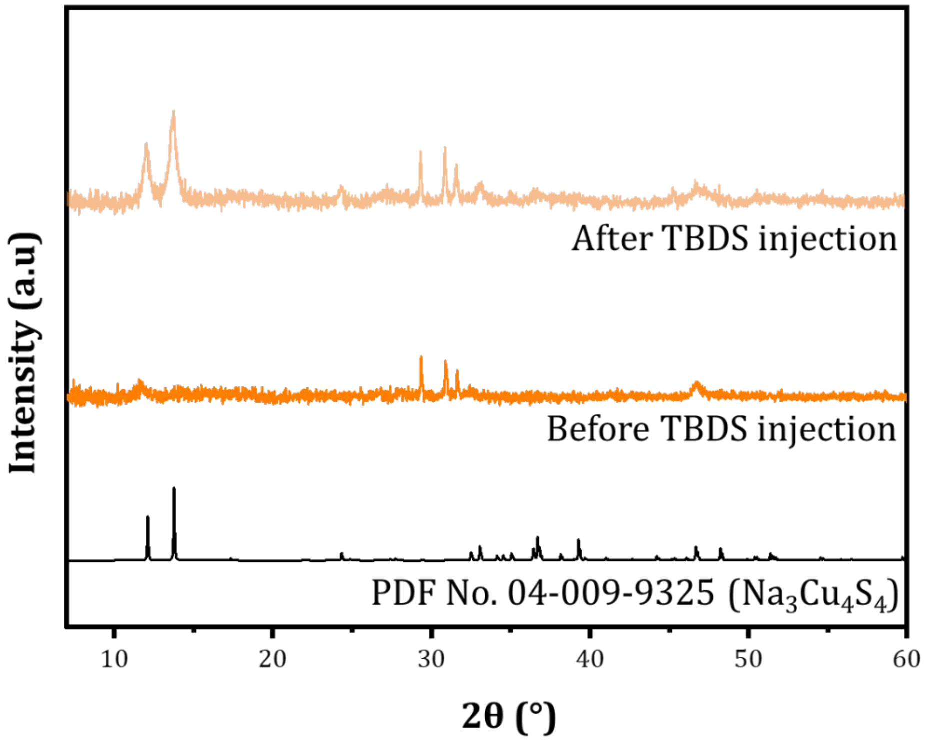

Figure S13 To avoid the formation of copper sulfide which occurs in the heat up phase, the copper precursor was added to a solution of Na-oleate at the reaction temperature (320°C). The temperature was allowed to return to 320°C before the sulfur injection was carried out and the results yield $Na_3Cu_4S_4$ formation without Cu_9S_5 forming first. The peaks in the graph before the TBDS injection could not be matched with any XRD pattern in the database can be attributed to a complex framework which forms between Na-Oleate and Cu-DDTC.

7.2 XRD of control to show $Na_3Cu_4S_4$ does not transform into $Na_2Cu_4S_3$

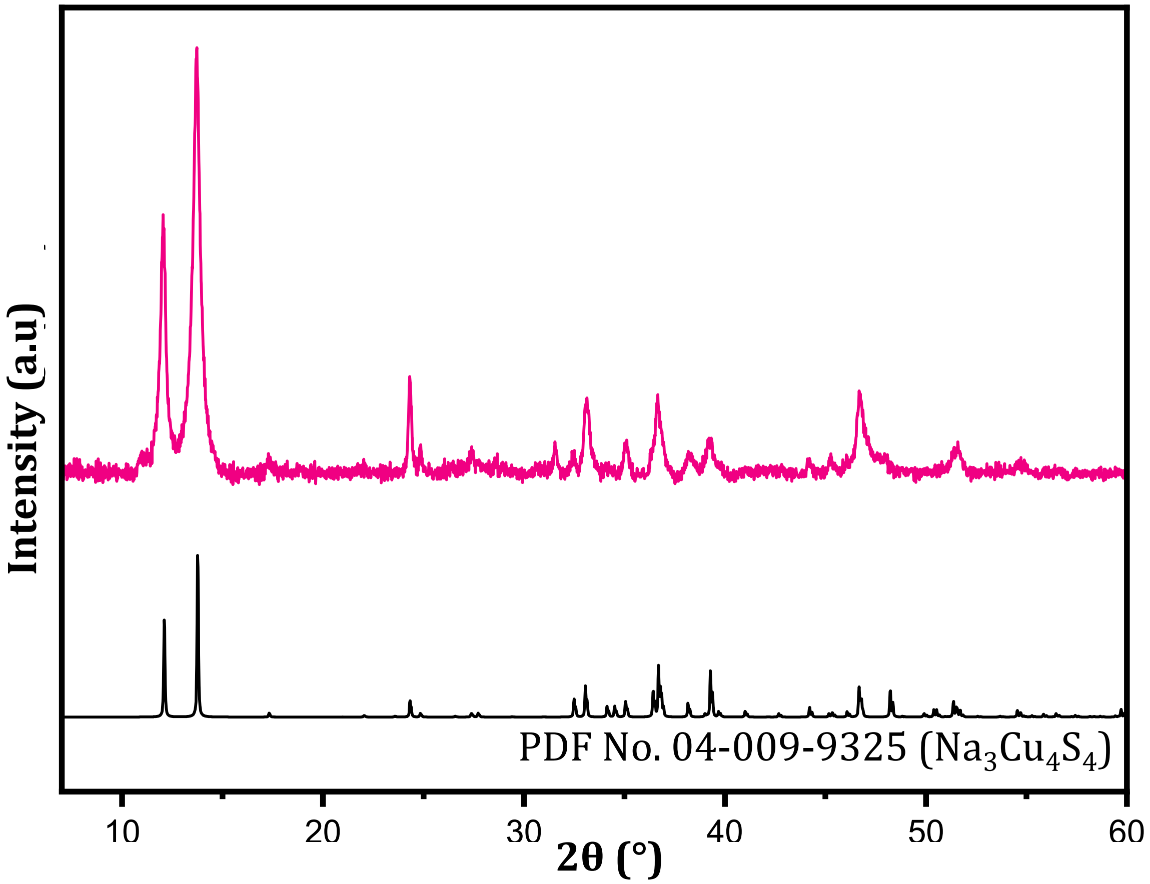

Figure S14. $Na_3Cu_4S_4$ was allowed to form and then 1-DDT was added to the reaction solution to determine if it would cause a transformation into the metastable $Na_2Cu_4S_3$. The XRD shows the transformation does not occur and the two structures are not interchangeable through crystallographic transformation.

7.3 XRD analysis of control experiments for $Na_2Cu_4S_3$

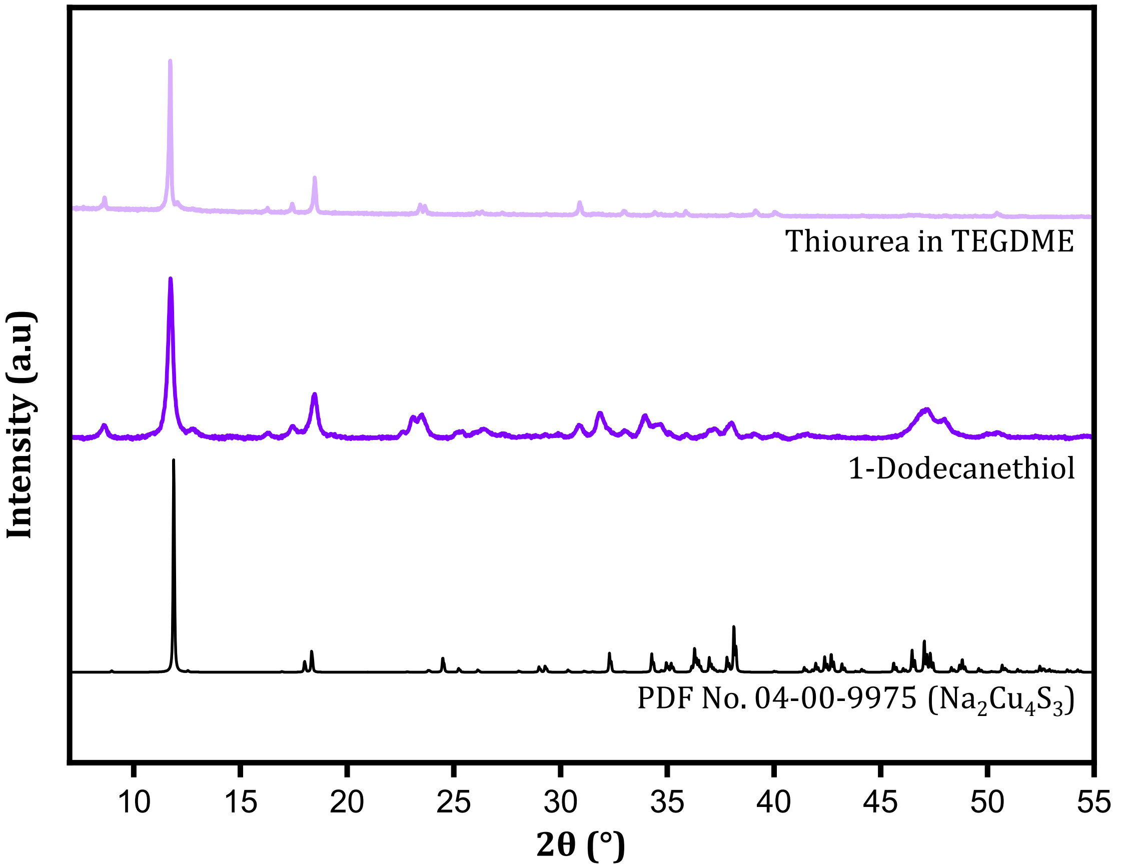

Figure S15 XRD of control reactions with variation in sulfur precursor for $Na_2Cu_4S_3$. 8mmol of thiourea was dissolved in 8ml TEGDME and used as the sulfur injection.

7.4 XRD analysis of control experiments for $Na_3Cu_4S_4$

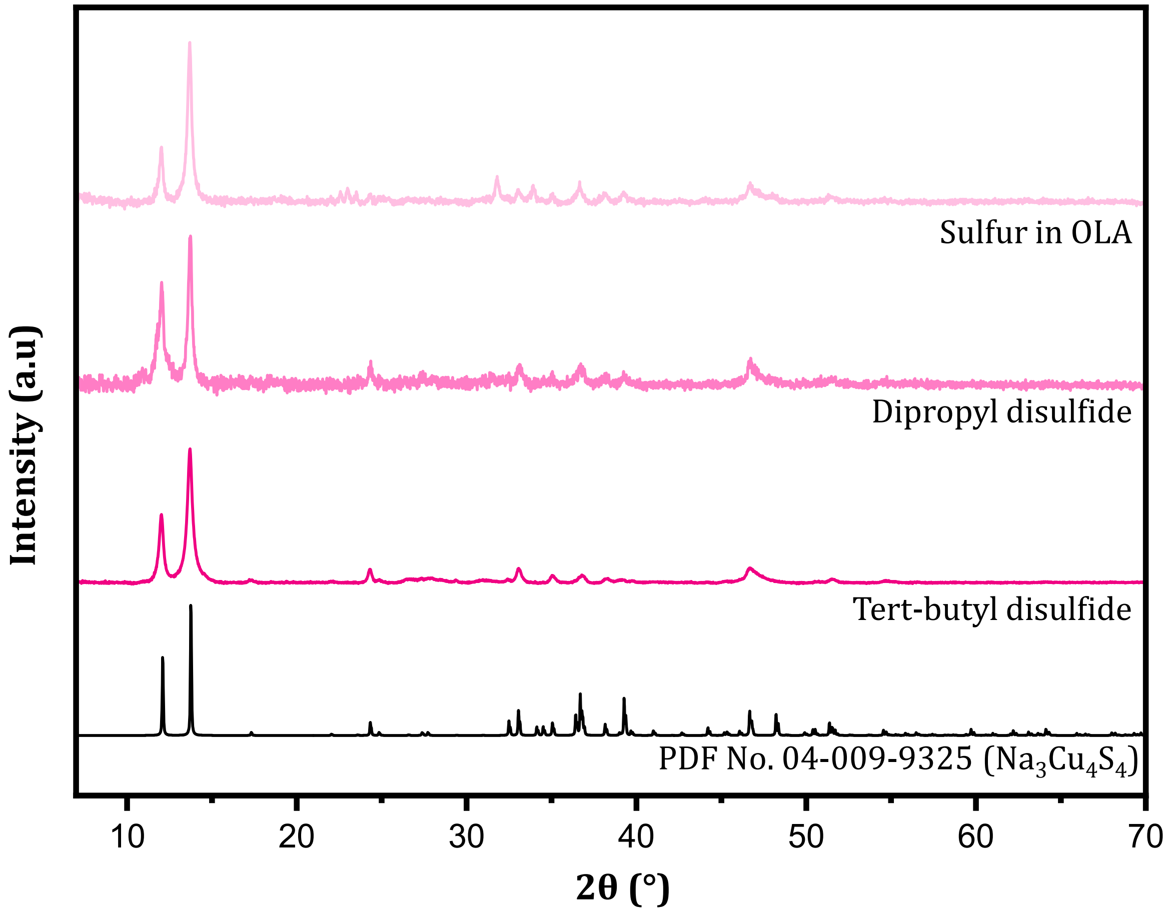

Figure S16 XRD of control reactions with variation in sulfur precursor for $Na_3Cu_4S_4$.

7.5 TEM images of control experiments for $Na_3Cu_4S_4$

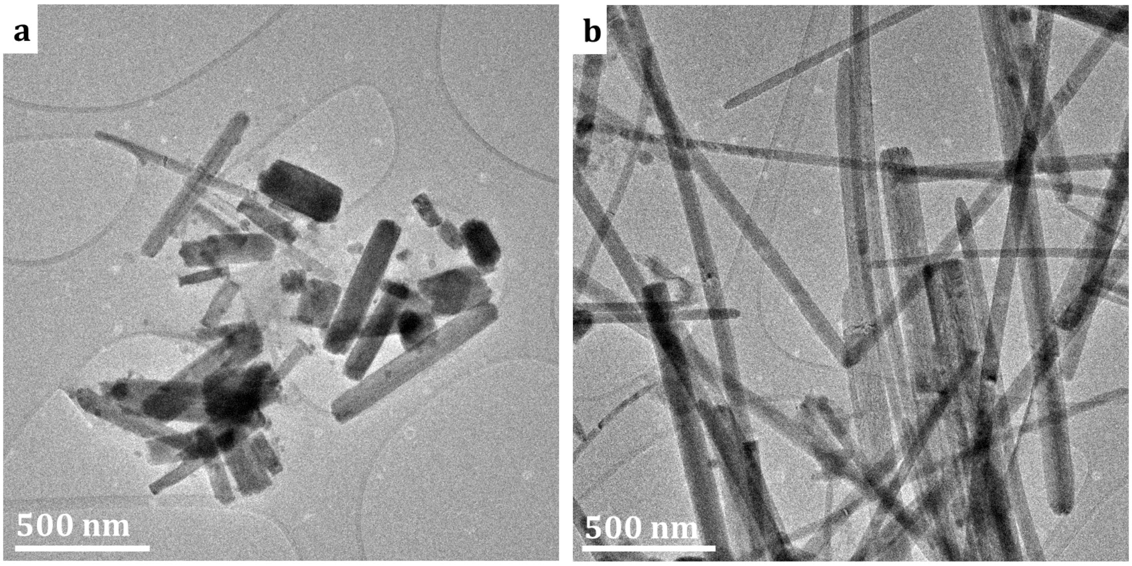

Figure S17 TEM of control reactions for $Na_3Cu_4S_4$ (a) propyl disulfide (b) Sulfur in OLA.

8. Analysis Methods

8.1 X-ray Diffraction (XRD)

All XRD patterns were taken by preparing a drop-cast film of the sample on a p-type boron-doped silicon zero background. The XRD experiments were conducted using a PANalytical Empyrean instrument equipped with a Cu Kα radiation source (λ= 1.5418 Å) and a 1-D X'celerator strip detector with a diffractometer operating at 40 kV and 40 mA.

8.2 Rietveld Refinement

XRD patterns were analysed using Rietveld refinement in Jana2006 software to determine the goodness of fit of experimental patterns with known crystal structures from the PDF database. Lattice parameters and weighted-profile R-factor. For aliquots, only lattice and profile parameters were refined; structures were used exactly as described in the corresponding CIF files.

8.3 Electron Microscopy

For transmission electron microscopy (TEM) nanocrystal samples were prepared by dispersion in toluene and drop casting on continuous carbon coated 200 mesh nickel grids. Low resolution and high-resolution TEM (HRTEM) and dark-field scanning transmission electron microscopy (DFSTEM) were carried out using a 200 kV JEOL JEM-2100F field emission microscope, equipped with a Gatan Ultra scan CCD camera and EDAX Genesis energy dispersive x-ray spectroscopy (EDS) detector. The analysis of interplanar distances of HRTEM was carried out using GMS3 software from the selected area FFT.

8.4 X-ray Photoelectron Spectroscopy (XPS)

XPS spectra were measured using a Kratos AXIS ULTRA spectrometer fitted with a mono Al Kα (1486.58eV) X-ray gun. Calibration was performed using the C 1s line at 284.8 eV, while

construction and peak fitting were performed using CasaXPS software. Samples were prepared for XPS by drop-cast film on a p-type boron-doped silicon.

8.5 Fourier Transform Infrared Spectroscopy

FTIR spectra were measured using an Agilent-Cary 630 FTIR. A transmission FTIR module was used on the instrument. The sample was dispersed in toluene and sandwiched between 2 x KBr salt plates. The plates were then secured in a sample holder and placed in the transmission module.

8.6 Computational Methods

All density functional theory (DFT) calculations were performed using the projector augmented wave method as implemented in the Vienna Ab initio Simulation Package (VASP).[3–5] The plane-wave cutoff energy and k-mesh density were converged to a total energy difference of 1 meV/atom, with a 30% increase in plane-wave cutoff energy during relaxations to avoid Pulay stress effects. For all calculations the HSE06 hybrid DFT functional was used which combines 75% exchange and 100% of the correlation energy from the PBE semi-local functional with 25% exact Hartree–Fock (HF) exchange at short ranges.[6–8] The upper limits to the photovoltaic efficiency were determined from the HSE06 electronic and optical calculations, using the 'Spectroscopic Limited Maximum Efficiency' (SLME).[9] The orbital decomposed density of states were calculated using the Lobster package[10] and band structures were plotted using the SUMO package.[11]

References

(1) Ivanova, T. M.; Maslakov, K. I.; Sidorov, A. A.; Kiskin, M. A.; Linko, R. V.; Savilov, S. V.; Lunin, V. V.; Eremenko, I. L. XPS Detection of Unusual Cu(II) to Cu(I) Transition on the Surface of Complexes with Redox-Active Ligands. *J Electron Spectros Relat Phenomena* **2020**, *238*, 146878. https://doi.org/10.1016/j.elspec.2019.06.010.

(2) Peplinski, Z.; Brown, D. B.; Watt, T.; Hatfield, W. E.; Daylc, P. Contribution from the Electrical Properties of Na3Cu4S4, a Mixed-Valence One-Dimensional Metal. *Inorg. Chem* **1982**, *21*, 1752–1755.

(3) Kresse, G.; Hafner, J. Ab Initio Molecular Dynamics for Liquid Metals. *Phys Rev B* **1993**, *47* (1), 558–561. https://doi.org/10.1103/PhysRevB.47.558.

(4) Kresse, G.; Furthmüller, J. Efficient Iterative Schemes for Ab Initio Total-Energy Calculations Using a Plane-Wave Basis Set. *Phys Rev B* **1996**, *54* (16), 11169–11186. https://doi.org/10.1103/PhysRevB.54.11169.

(5) Kresse, G.; Joubert, D. From Ultrasoft Pseudopotentials to the Projector Augmented-Wave Method. *Phys Rev B* **1999**, *59* (3), 1758–1775. https://doi.org/10.1103/PhysRevB.59.1758.

(6) Heyd, J.; Scuseria, G. E.; Ernzerhof, M. Hybrid Functionals Based on a Screened Coulomb Potential. *J Chem Phys* **2003**, *118* (18), 8207–8215. https://doi.org/10.1063/1.1564060.

(7) Krukau, A. V.; Vydrov, O. A.; Izmaylov, A. F.; Scuseria, G. E. Influence of the Exchange Screening Parameter on the Performance of Screened Hybrid Functionals. *J Chem Phys* **2006**, *125* (22). https://doi.org/10.1063/1.2404663.

(8) Perdew, J. P.; Burke, K.; Wang, Y. Generalized Gradient Approximation for the Exchange-Correlation Hole of a Many-Electron System. *Phys Rev B* **1996**, *54* (23), 16533–16539. https://doi.org/10.1103/PhysRevB.54.16533.

(9) Yu, L.; Zunger, A. Identification of Potential Photovoltaic Absorbers Based on First-Principles Spectroscopic Screening of Materials. *Phys Rev Lett* **2012**, *108* (6), 068701. https://doi.org/10.1103/PhysRevLett.108.068701.

(10) Deringer, V. L.; Tchougréeff, A. L.; Dronskowski, R. Crystal Orbital Hamilton Population (COHP) Analysis As Projected from Plane-Wave Basis Sets. *J Phys Chem A* **2011**, *115* (21), 5461–5466. https://doi.org/10.1021/jp202489s.

(11) M Ganose, A.; J Jackson, A.; O Scanlon, D. Sumo: Command-Line Tools for Plotting and Analysis of Periodic Ab Initio Calculations. *J Open Source Softw* **2018**, *3* (28), 717. https://doi.org/10.21105/joss.00717.
